# The RNA-binding protein ProQ directly binds and regulates virulence genes in enterohemorrhagic *Escherichia coli* O157:H7

**DOI:** 10.3389/fcimb.2025.1627518

**Published:** 2025-08-29

**Authors:** Ye Qian, Chenguang Zheng, Runhua Han

**Affiliations:** ^1^ School of Basic Medical Sciences, North China University of Science and Technology, Tangshan, China; ^2^ School of Public Health, North China University of Science and Technology, Tangshan, China; ^3^ Department of Chemistry, University of Manitoba, Winnipeg, MB, Canada

**Keywords:** RNA regulation, EHEC, O157:H7, RNA binding protein, ProQ, small regulatory RNA, virulence gene regulation, LEE

## Abstract

ProQ has recently emerged as a major post-transcriptional regulator in bacteria through directly binding to diverse mRNAs and small regulatory RNAs (sRNAs). However, the impact of ProQ in many pathogenic bacteria remains poorly understood. In this study, we investigated the role of ProQ in enterohaemorrhagic *Escherichia coli* (EHEC), a non-invasive intestinal pathogen. We found that deletion of *proQ* significantly enhanced cell adherence ability of EHEC and led to upregulation of the locus of enterocyte effacement (LEE) pathogenicity island. This effect was accompanied by reduced expression of genes encoding known LEE regulators, including protein factors (*ihfA* and *hns*) and sRNAs (GlmY and GlmZ), resulting from decreased stability of their transcripts in the absence of *proQ*. Additionally, *proQ* positively regulated bacterial motility by stabilizing *fliC* mRNA. We further demonstrated that ProQ directly binds to *ihfA*, *hns*, *glmY*/*glmZ* and *fliC* transcripts at secondary structures located near their 3’ ends. Beyond virulence regulation, ProQ also contributed to antibiotic persistence of EHEC and its survival under host-associated stress conditions. Collectively, our findings highlight ProQ as a key gene regulator in EHEC, providing new insights into how this pathogen modulates its virulence at the post-transcriptional level.

## Introduction

Post-transcriptional regulation is a crucial mechanism that allows bacteria to rapidly adapt to changing environmental conditions by modulating gene expression at the RNA level (Van Assche et al., 2015). Small regulatory RNAs (sRNAs) play a central role in this process, acting as versatile regulators that influence mRNA stability, translation, and protein activity (Felden and Augagneur, 2021; Papenfort and Melamed, 2023). sRNAs primarily exert their effects through direct base-pairing with target mRNAs or by interacting with proteins (Storz et al., 2011). These interactions enable bacteria to fine-tune gene expression in response to diverse stresses, such as nutrient fluctuations, quorum sensing signals, and virulence-related cues (Papenfort and Melamed, 2023). A critical aspect of sRNA-mediated regulation is its dependence on RNA-binding proteins (RBPs). These proteins function as molecular chaperones or sequestration agents, stabilizing sRNAs or facilitating their interactions with target mRNAs (Van Assche et al., 2015). Together, sRNAs and their associated binding proteins form intricate regulatory networks that enhance bacterial fitness, survival, and pathogenicity.

ProQ is a widely conserved sRNA-binding protein in many bacteria that plays a pivotal role in post-transcriptional regulation (Smirnov et al., 2016; Olejniczak and Storz, 2017; Liao and Smirnov, 2023). As a member of the FinO-domain family of RNA chaperones, ProQ has emerged as a key player in facilitating RNA-mediated gene regulation. Recent high-throughput techniques, such as CLIP-seq (cross-linking and immunoprecipitation followed by sequencing) or RIL-seq (RNA interaction by ligation and sequencing), have mapped ProQ’s RNA targets across several bacterial species, revealing its broad regulatory roles (Holmqvist et al., 2018; Melamed et al., 2020; Bergman et al., 2024). In *Escherichia coli* and *Salmonella typhimurium*, ProQ binds hundreds of RNAs with a preference for highly structured RNAs, including sRNAs and 3’ untranslated regions (UTRs) of mRNAs (Holmqvist et al., 2018). It promotes the sRNA-mRNA pairing and contributes to the sRNA stabilization, as well as the regulation of mRNA stability (Smirnov et al., 2017; Holmqvist et al., 2018; Bergman et al., 2025). Functional studies have shown that *proQ* mutant strains exhibit defects in stress adaptation, reduced virulence, impaired antibiotic resistance, and altered motility, underscoring the critical role of ProQ in bacterial physiology (Westermann et al., 2019; Bauriedl et al., 2020; Rizvanovic et al., 2022; Feng et al., 2024; Ghandour et al., 2025; Mihaita et al., 2025). For example, ProQ contributes to the expression of virulence factors encoded in *Salmonella* pathogenicity islands, enhancing the bacteria’s ability to invade host cells and evade the immune system (Westermann et al., 2019). In *Neisseria meningitidis*, ProQ stabilizes sRNAs and mRNAs that are critical for bacterial survival and pathogenesis, including those involved in adhesion, immune evasion, and iron uptake (Bauriedl et al., 2020). By controlling these processes, ProQ supports the adaptability and pathogenic success of these microorganisms. The essential functions of ProQ also highlight its potential—and that of its associated pathways—as promising targets for the development of novel antimicrobial strategies. However, despite these insights, the contribution of ProQ to bacterial pathogenicity remains incompletely understood, particularly in other clinically relevant pathogens.

Enterohemorrhagic *Escherichia coli* (EHEC) is a significant foodborne pathogen that poses a serious public health threat worldwide (Correa-Martinez et al., 2022). EHEC infections can lead to a spectrum of gastrointestinal diseases, ranging from mild diarrhea to severe conditions such as hemorrhagic colitis and life-threatening hemolytic uremic syndrome (HUS) (Pacheco and Sperandio, 2012). Among its serotypes, EHEC O157:H7 is the most notorious, having been implicated in numerous outbreaks often linked to the consumption of contaminated food or water, as well as contact with infected animals or their environments (Connolly et al., 2015). A defining feature of EHEC O157:H7 infection is its ability to adhere tightly to the intestinal epithelium, a process mediated by virulence factors encoded within the locus of enterocyte effacement (LEE) pathogenicity island. This intimate interaction results in the formation of attaching and effacing (A/E) lesions, a hallmark of EHEC pathogenesis (Connolly et al., 2015). Furthermore, flagella play a critical role in the lifecycle and pathogenicity of EHEC O157:H7. These helical structures, composed primarily of the protein flagellin, provide motility, enabling EHEC to navigate its environment and reach optimal colonization sites in the gastrointestinal tract (Sun et al., 2022a).

The expression of EHEC O157:H7 virulence factors can activate the host innate immune system during infection. Therefore, the regulation of these factors must be tightly orchestrated, allowing EHEC O157:H7 to balance its pathogenesis and immune evasion in response to microenvironmental cues within the host. EHEC O157:H7 achieves this through a highly coordinated system that integrates genetic, environmental, and host-derived signals to control the expression of virulence factors involved in motility, adhesion, toxin production, and immune evasion (Connolly et al., 2015; Woodward et al., 2019; Liu et al., 2020; Wale et al., 2021). Notably, post-transcriptional regulation mediated by sRNAs and RBPs is crucial for EHEC O157:H7 to synchronize its virulence, stress responses, and adaptation to the host environment (Shakhnovich et al., 2009; Gruber and Sperandio, 2014; Han et al., 2017, 2024; Wang et al., 2017; Sudo et al., 2018; Melson and Kendall, 2019; Sauder and Kendall, 2021). For example, Hfq, a well-characterized RBP, enhances the stability and activity of several sRNAs in EHEC O157:H7, facilitates their pairing with target mRNAs, and influences processes such as virulence gene expression and stress resistance (Shakhnovich et al., 2009; Tree et al., 2014; Waters et al., 2017). Hfq can also directly bind to LEE genes independently of sRNAs, modulating their expression (Sudo et al., 2022). Another major RBP, CsrA, directly or indirectly regulates genes involved in adhesion, motility, biofilm formation, as well as quorum sensing and various metabolic pathways in EHEC O157:H7 (Wang et al., 2017; Ye et al., 2018; Sun et al., 2022b). In contrast, the role of ProQ in EHEC O157:H7 pathogenesis and stress response remains poorly understood. Understanding how ProQ contributes to these processes could provide new insights into the regulatory mechanisms driving EHEC O157:H7 virulence and adaptation.

In this study, we uncovered the direct binding of ProQ to several virulence-related mRNAs and sRNAs in EHEC O157:H7. Additionally, the contribution of ProQ to antibiotic persistence and acclimation to various infection-related stresses was also identified. Our findings thus highlight critical roles of understudied RNA-binding proteins in EHEC.

## Results

### Expression of *proQ* is essential for the survival of EHEC O157:H7 under host-derived stresses

In the EHEC O157:H7 EDL933 strain, the *proQ* gene is expressed from the minus strand and located between *z2879* (encoding a hypothetical protein) and *prc* (encoding a periplasmic protease) (**Supplementary Figure S1A**). To investigate the cellular role of *proQ* in EHEC O157:H7, a *proQ* deletion strain (Δ*proQ*) was constructed. Given that the promoter region of *prc* starts within the *proQ* coding sequence (Kerr et al., 2014), only the first 190 nucleotides of *proQ* gene (the region upstream the *prc* promoter start site) were removed to prevent the disruption of *prc* promoter. Consistent with this design, the level of *prc* transcript remained unchanged in the Δ*proQ* strain compared to wild type (WT) (**Supplementary Figure S2**), suggesting that the partial deletion of the *proQ* sequence did not affect *prc* promoter activity. Moreover, Δ*proQ* exhibited comparable growth to WT in LB medium (pH 7.2) at 37°C (**Supplementary Figure S1B**), indicating a dispensable role of *proQ* in EHEC O157:H7 fitness under normal conditions.

**Figure 1:**
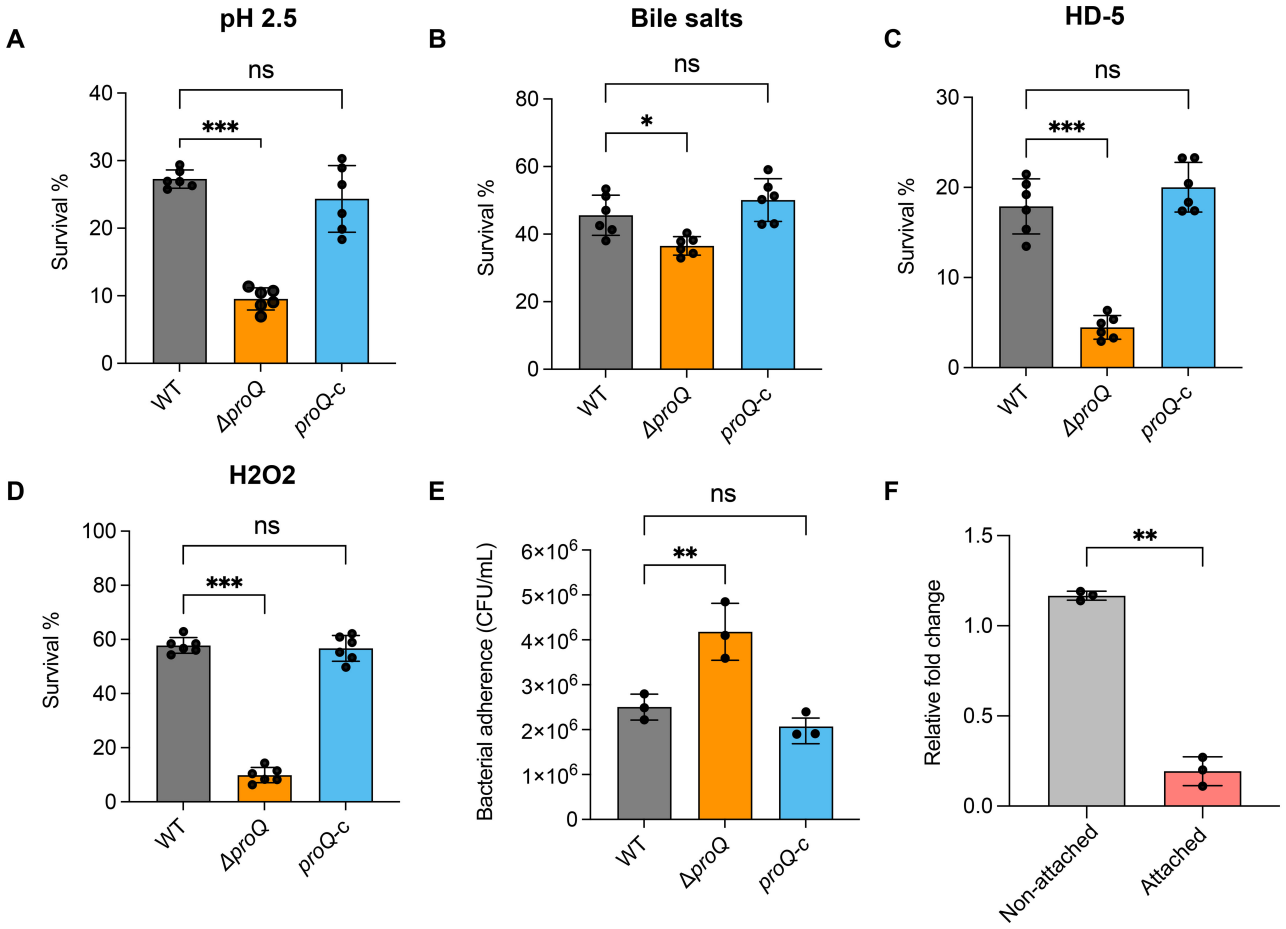
ProQ expression is essential for stress acclimation and bacterial adherence ability of EHEC O157:H7. **(A)** Survival of WT, Δ*proQ*, and *proQ*-c strains after incubation in acidified LB medium (pH 2.5) for 30 min. **(B)** Survival of WT, Δ*proQ*, and *proQ*-c strains after treatment of 2% bile salts for 1 h. **(C)** Survival of WT, Δ*proQ*, and *proQ*-c strains after incubation with 25 µM human defensin 5 (HD-5) for 1 h. **(D)** Survival of WT, Δ*proQ*, and *proQ*-c strains after incubation with 5 mM H_2_O_2_ for 30 min. **(E)** Adherence of WT, Δ*proQ*, and *proQ*-c strains to Caco-2 cells after 3 h of incubation. **(F)** qRT-PCR analysis of *proQ* level in WT before and after adherence to Caco-2 cells. WT, EHEC O157:H7 wild-type strain; Δ*proQ*, *proQ* deletion strain; *proQ*-c, *proQ* complementation strain. The average values from six **(A–D)** or three **(E, F)** biological replicates with SD are shown. Statistical significance was assessed using one-way ANOVA analysis **(A–E)**, or two-tailed Student’s t-test **(F)**. *, *P* ≤ 0.05; **, *P* ≤ 0.01; ***, *P* ≤ 0.001; ns, non-significant.

EHEC O157:H7 must withstand the harsh conditions of the stomach and small intestine before adhering to intestinal epithelial cells to initiate colonization in the large intestine (Connolly et al., 2015). To test the role of *proQ* in these environments, we challenged WT, Δ*proQ*, and complemented (*proQ*-c) strains with low pH (2.5), 1% bile salts, and 25 μM human defensin-5 (HD-5), simulating stomach and small intestine conditions. In all cases, deletion of *proQ* significantly impaired bacterial survival, and this defect was rescued by expressing *proQ* from a low-copy plasmid under the control of its native promoter (**Figures 1A–C**). These results highlight the importance of *proQ* expression for survival in stomach- and small intestine-like environments before entering the large intestine. Moreover, adherence of EHEC O157:H7 to follicle-associated epithelium in the intestine brings the bacteria into contact with macrophages, where they encounter oxidative stress (Etienne-Mesmin et al., 2011). To determine if *proQ* also contributes to oxidative stress resistance of EHEC O157:H7, we exposed the WT, Δ*proQ* and *proQ*-c strains to 5 mM H_2_O_2_ for 30 minutes. We observed that survival of Δ*proQ* was significantly reduced compared to WT and *proQ*-c strains after H_2_O_2_ treatment (**Figure 1D**). Altogether, our data suggest that *proQ* expression is required for EHEC O157:H7 to survive in a range of adverse environment in host gastrointestinal tract, and to establish a successful infection eventually.

### ProQ negatively affects adherence ability of EHEC O157:H7 to colonic epithelial cells

To further assess the role of ProQ in the virulence of EHEC O157:H7, WT, Δ*proQ*, and *proQ*-c strains were grown to mid-log phase (OD_600nm_ = 0.6), incubated with Caco-2 cells, and their adherence abilities were compared. We observed that the Δ*proQ* strain exhibited significantly higher adherence to Caco-2 cells compared to WT (**Figure 1E**). Notably, *prc* is also required for robust translocation of EspF, one of the LEE effectors, which could potentially contribute to the virulence phenotype of the Δ*proQ* strain. However, the expression of this gene was not affected by *proQ* deletion (**Supplementary Figure S2**). This suggests that the increased adherence observed in the Δ*proQ* strain is primarily due to the loss of *proQ* expression rather than indirect effects from a disrupted *prc* expression. Interestingly, we also found that the expression of the *proQ* gene was significantly lower in attached EHEC O157:H7 cells compared to non-attached cells (**Figure 1F**), indicating that *proQ* is downregulated following cell adherence. Together, these findings suggest that *proQ* acts as a negative regulator of EHEC O157:H7 adherence to host cells.

### ProQ modulates expression of LEE genes at both transcriptional and post-transcriptional levels

**Figure 2:**
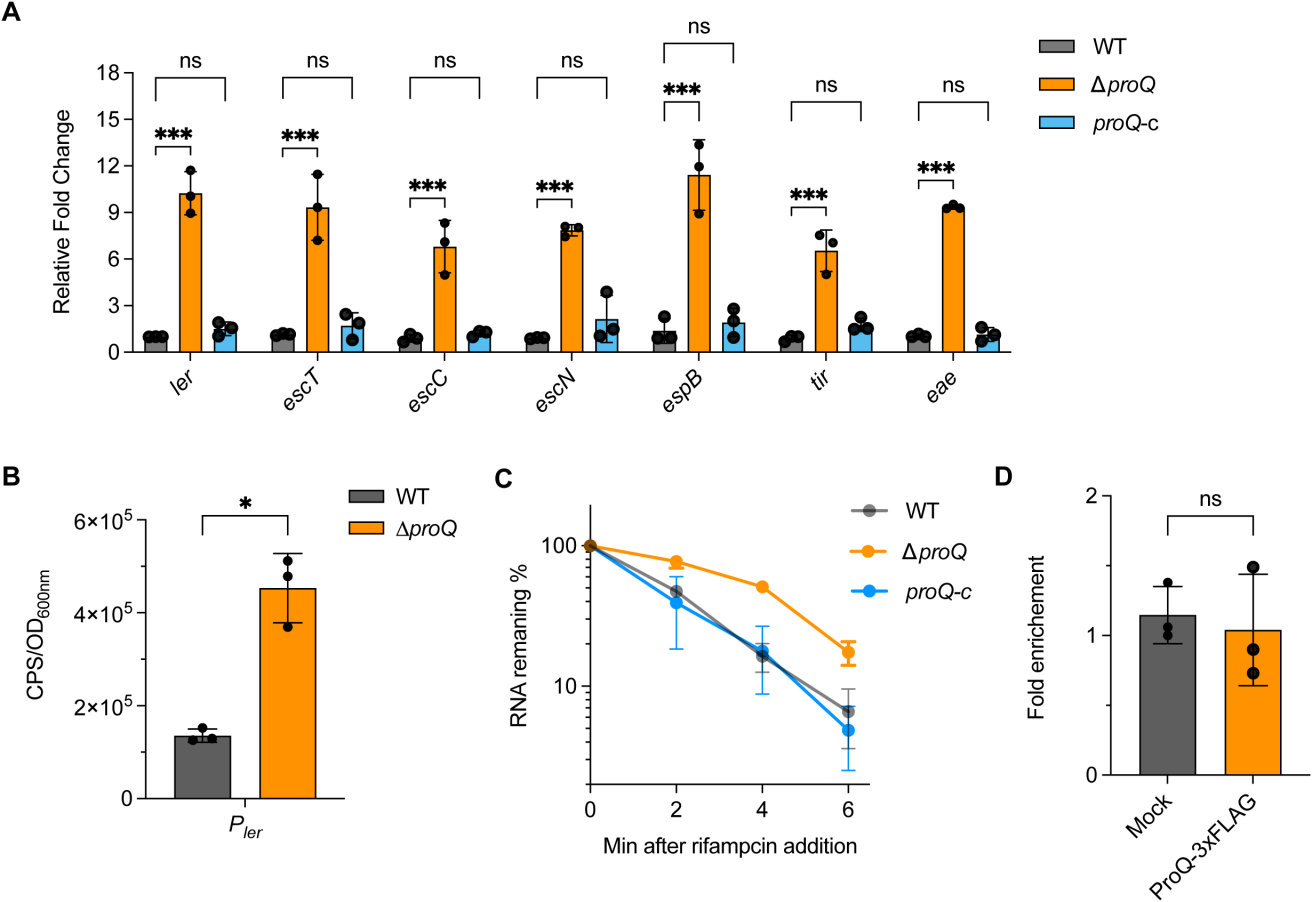
ProQ modulates expression of LEE genes. **(A)** qRT-PCR analysis of LEE gene expression in WT, Δ*proQ*, and *proQ*-c strains. **(B)** Promoter activity of *ler* in WT and Δ*proQ* strains, measured using a transcriptional reporter assay. **(C)** Stability of *ler* in WT, Δ*proQ*, and *proQ*-c strains. Bacterial cultures were grown to an OD_600nm_ of 0.6, rifampicin was added to inhibit transcription, and qRT-PCR was performed at the indicated time points to assess *ler* mRNA levels. **(D)** *In vivo* binding of ProQ to *ler* mRNA. RNA associated with ProQ-3×FLAG was purified by RNA co-immunoprecipitation (co-IP) from an EHEC O157:H7 strain expressing ProQ-3×FLAG, and enrichment of *ler* was assessed by qRT-PCR. WT, EHEC O157:H7 wild-type strain; Δ*proQ*, *proQ* deletion strain; *proQ*-c, *proQ* complementation strain. The average values from three biological replicates with SD are shown. Statistical significance was assessed via two-way ANOVA analysis **(A)**, or two-tailed Student’s t-test **(B, D)**. *, *P* ≤ 0.05; ***, *P* ≤ 0.001; ns, non-significant.

Given that the LEE locus is one of the most critical virulence factors for EHEC O157:H7 colonization, we next investigated whether ProQ affects its expression. Using qRT-PCR, we compared the expression of representative genes from the seven LEE operons [*ler* (LEE1), *escT* and *escC* (LEE2), *escN* (LEE3), *espB* (LEE4), *eae* and *tir* (LEE5)] across different *proQ* genetic backgrounds. As shown in **Figure 2A**, the expression of all tested LEE genes was significantly upregulated in the Δ*proQ* strain and returned to WT levels upon *proQ* complementation. Consistently, EspB secretion increased markedly in the absence of *proQ* and was restored by *proQ* complementation (**Supplementary Figure S3**), indicating that *proQ* negatively regulates T3SS-mediated secretion of LEE-encoded effectors. Notably, the upregulation of the master regulator *ler* in the Δ*proQ* strain suggests that ProQ may indirectly affect other LEE genes through *ler*. To further dissect how ProQ regulates *ler* expression, we fused the *ler* promoter to a *lux* reporter gene and measured its activity in WT and Δ*proQ* strains. We observed an enhanced *ler* promoter activity in the Δ*proQ* background (**Figure 2B**), suggesting a transcriptional regulation of *ler* by ProQ. Given that ProQ is also known to function post-transcriptionally, we examined whether ProQ affects *ler* mRNA stability. RNA decay assays revealed increased *ler* transcript stability in the Δ*proQ* strain compared to WT and *proQ* complementation strains (**Table 1**; **Figure 2C**), indicating that ProQ regulates *ler* at both transcriptional and post-transcriptional levels. We then conducted an RNA co-immunoprecipitation (RIP) assay to determine whether ProQ directly binds *ler* mRNA *in vivo*. Using a chromosomally 3xFLAG-tagged ProQ strain, we immunoprecipitated associated RNAs and assessed *ler* enrichment by qRT-PCR. Interestingly, no enrichment of *ler* mRNA was detected in the ProQ-3xFLAG sample (**Figure 2D**), suggesting that ProQ does not directly bind *ler* mRNA and the regulation of *proQ* on *ler* is likely mediated by other factors.

**Table 1:** Half-lives of virulence-associated mRNAs and sRNAs in EHEC O157:H7 WT, *proQ* deletion (Δ*proQ*) and complementation (*proQ*-c) strains, measured by qRT-PCR.

Name	Type	WT/min	Δ*proQ*/min	*proQ*-c/min
*ler*	mRNA	1.99 ± 0.27	4.13 ± 0.50	1.95 ± 1.12
*hns*	mRNA	13.67 ± 2.78	4.59 ± 1.29	10.77 ± 1.31
*ihfA*	mRNA	1.89 ± 0.59	0.94 ± 0.10	4.29 ± 1.04
*fliC*	mRNA	2.55 ± 0.32	1.59 ± 0.43	3.83 ± 0.92
GlmY	sRNA	1.75 ± 0.42	0.57 ± 0.06	3.19 ± 2.54
GlmZ	sRNA	1.00 ± 0.09	0.59 ± 0.06	1.02 ± 0.31

The average values from three biological replicates with SD are shown.

### ProQ regulates LEE transcriptional regulators *ihfA* and *hns* posttranscriptionally

**Figure 3:**
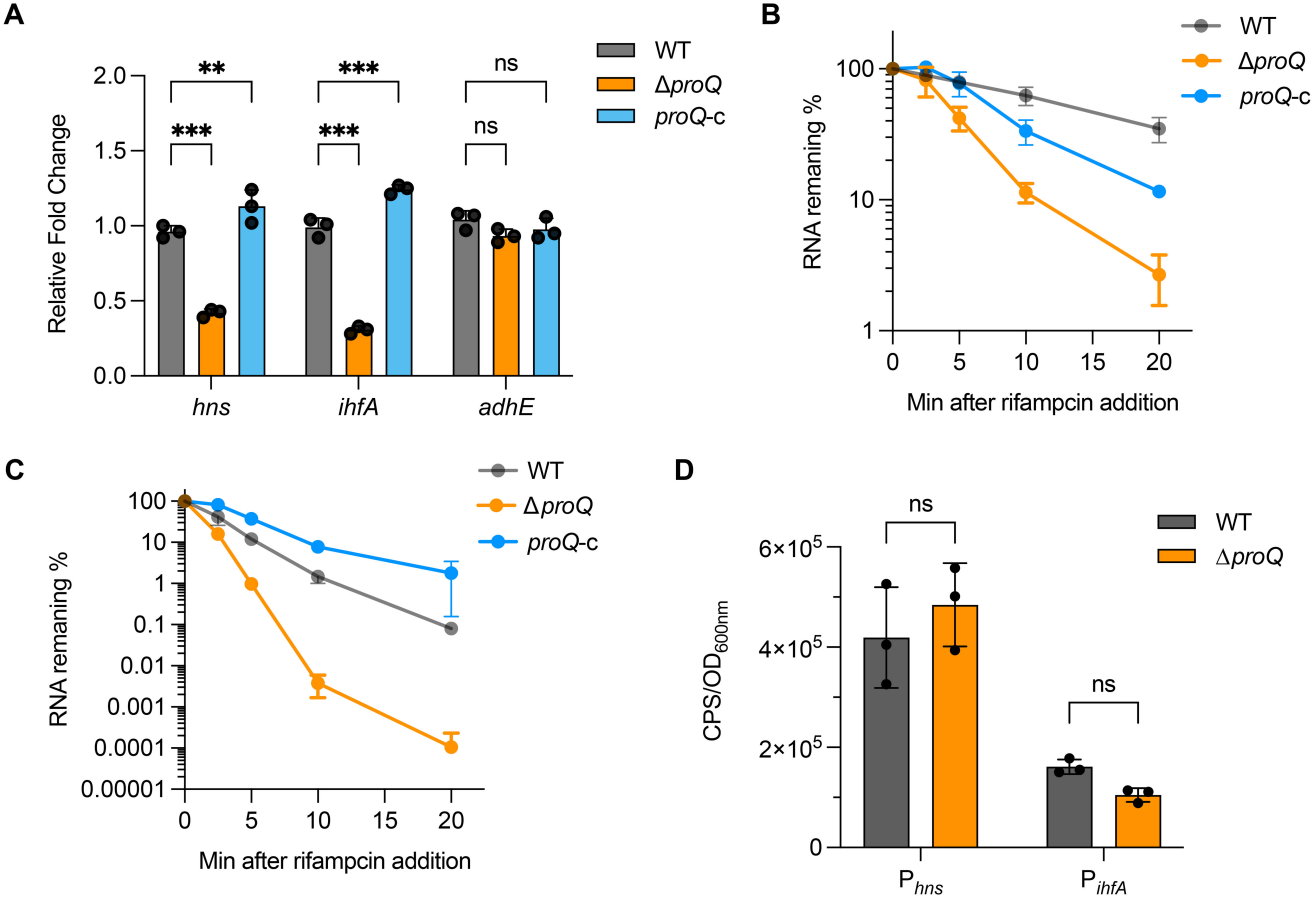
ProQ stabilizes LEE transcriptional regulator genes. **(A)** Expression of *ihfA*, *hns*, and *adhE* in WT, Δ*proQ*, and *proQ*-c strains, measured by qRT-PCR. B-C. Stability of *hns* **(B)** and *ihfA* **(C)** in WT, Δ*proQ*, and *proQ*-c strains, measured by qRT-PCR. **(D)** Promoter activity of *ihfA* and *hns* in WT and Δ*proQ* strains, measured by transcriptional reporter assays. WT, EHEC O157:H7 wild-type strain; Δ*proQ*, *proQ* deletion strain; *proQ*-c, *proQ* complementation strain. The average values from three biological replicates with SD are shown. Statistical significance was assessed via two-way ANOVA analysis **(A)**, or two-tailed Student’s t-test **(D)**. **, *P* ≤ 0.01; ***, *P* ≤ 0.001; ns, non-significant.

The LEE expression was known to be modulated by multiple transcriptional regulators under various stresses (Woodward et al., 2019; Wale et al., 2021), providing potential indirect pathways for ProQ-mediated regulation on *ler*. Although little is known about how *proQ* affects gene expression in EHEC O157:H7, RNA-seq analysis has identified hundreds of genes whose expression is altered by *proQ* deletion or overexpression in the non-pathogenic *E. coli* K-12 strain (Melamed et al., 2020). Among these, three genes—*ihfA*, *hns*, and *adhE*—encode transcriptional regulators that are conserved in EHEC and known to modulate LEE expression through different mechanisms (Friedberg et al., 1999; Beckham et al., 2014; Levine et al., 2014). We then used qRT-PCR to assess whether these genes are affected by ProQ in EHEC O157:H7. Our results showed that deletion of *proQ* led to nearly a 2-fold decrease in *hns* expression and a 3-fold decrease in *ihfA* expression, both of which were restored by *proQ* complementation (**Figure 3A**). In contrast, *adhE* expression remained unchanged upon *proQ* deletion or complementation (**Figure 3A**). To further investigate the underlying mechanism, we examined *ihfA* and *hns* mRNA stability in the presence and absence of *proQ* expression. RNA decay assays revealed that both transcripts showed shorter half-lives in the Δ*proQ* strain compared to WT and *proQ* complementation (**Table 1**; **Figures 3B, C**). However, their promoter activities showed no differences between the two strains (**Figure 3D**), suggesting that ProQ only regulates *ihfA* and *hns* post-transcriptionally, which likely contributes to the observed changes in LEE expression and bacterial adherence in the *proQ* deletion strain.

### ProQ affects GlmY and GlmZ regulation in EHEC

**Figure 4:**
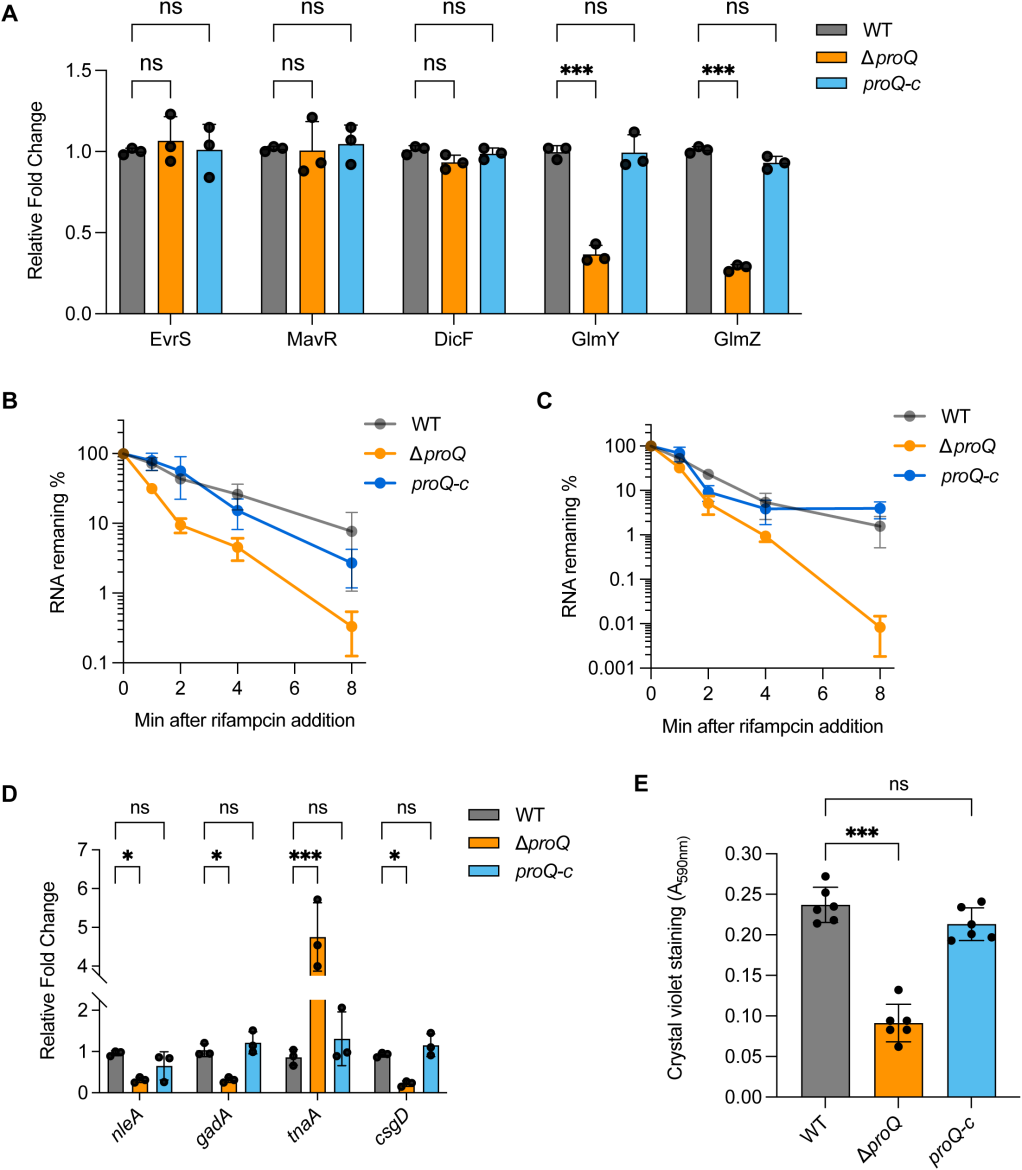
ProQ affects GlmY/Z regulation. **(A)** qRT-PCR analysis of GlmY, GlmZ, MavR, DicF and Esr41 levels in WT, Δ*proQ*, and *proQ*-c strains. **(B, C)**. Stability of GlmY **(B)** and GlmZ **(C)** in WT, Δ*proQ*, and *proQ*-c strains. Bacterial cultures were grown to an OD_600nm_ of 0.6, rifampicin was added to inhibit transcription, and qRT-PCR was performed at the indicated time points to assess the level of *glmY* and *glmZ* transcripts. **(D)** Expression of GlmY- and GlmZ-regulating genes in WT, Δ*proQ*, and *proQ*-c strains. **(E)** Biofilm formation in WT, Δ*proQ*, and *proQ*-c strains, assessed by crystal violet staining. WT, EHEC O157:H7 wild-type strain; Δ*proQ*, *proQ* deletion strain; *proQ*-c, *proQ* complementation strain. The average values from three **(A–D)** or six **(E)** biological replicates with SD are shown. Statistical significance was assessed via two-way ANOVA analysis. *, *P* ≤ 0.05; ***, *P* ≤ 0.001; ns, non-significant.

As an RNA binding protein, ProQ has been demonstrated to function as a chaperone to affect the regulation or stability of its sRNA targets (Smirnov et al., 2017; Melamed et al., 2020; Ghandour et al., 2025). We next investigated whether ProQ impacts sRNAs involved in LEE regulation in EHEC O157:H7. Using qRT-PCR, we analyzed the expression of five sRNAs, EvrS, DicF, MavR, GlmY and GlmZ, that were shown to influence LEE expression (Gruber and Sperandio, 2014; Sudo et al., 2018; Melson and Kendall, 2019; Sauder and Kendall, 2021; Han et al., 2024). Deletion of *proQ* markedly decreased the level of GlmY and GlmZ, whereas the level of the other sRNAs remained largely unchanged (**Figure 4A**). RNA decay assays showed that transcripts of GlmY and GlmZ were less stable in the Δ*proQ* strain (**Table 1**; **Figures 4B, C**). In contrast, complementation of *proQ* in the deletion strain restored their stability (**Table 1**; **Figures 4B, C**), suggesting that the ProQ protein protects these sRNAs from degradation. Consistent with these findings, deletion of *proQ* significantly altered the expression of several GlmY/GlmZ-regulated genes. Specifically, upon the deletion of *proQ*, the tryptophan synthesis gene *tnaA* was upregulated, while the acid resistance gene *gadA* and the non-LEE effector gene *nleA* were downregulated (**Figure 4D**). Expression of the curli formation regulatory gene, *csgD*, was also repressed when *proQ* was deleted (**Figure 4D**). To further investigate whether ProQ is involved in biofilm formation, crystal violet staining assays were performed. As shown in **Figure 4E**, the abolishment of *proQ* expression severely impaired biofilm formation, indicating that ProQ is a positive regulator of biofilm formation in EHEC O157:H7, which is consistent with the decreased *csgD* expression in Δ*proQ*. Altogether, these results show that ProQ stabilizes GlmY and GlmZ and further regulates various downstream genes and pathways controlled by these sRNAs.

### ProQ stabilizes *fliC* and regulates EHEC motility

**Figure 5:**
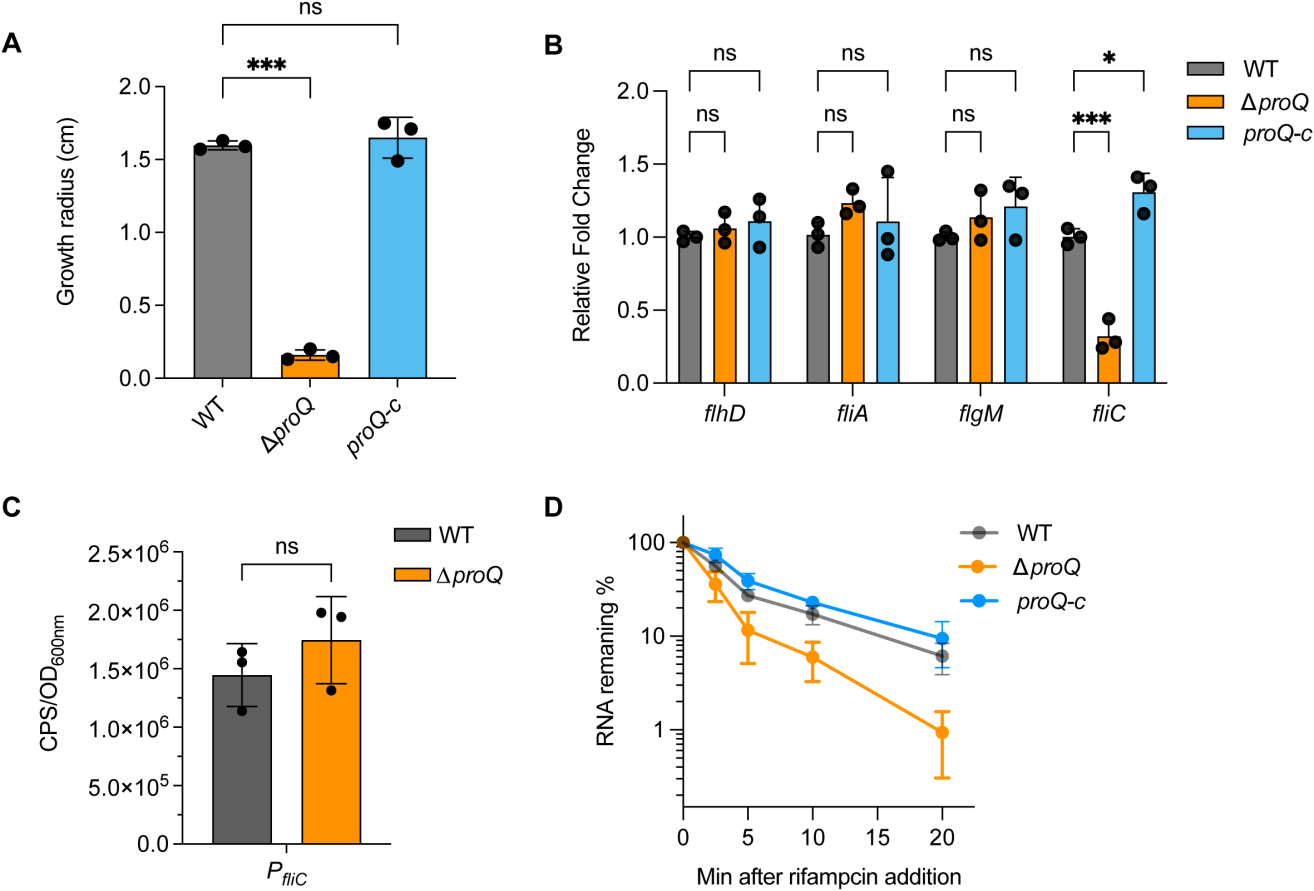
ProQ influences EHEC O157:H7 motility through stabilizing *fliC*. **(A)** Growth radius of WT, Δ*proQ*, and *proQ*-c strains after 10 h at 37°C on agar plates. **(B)** qRT-PCR analysis of *flhD*, *fliA, flgM* and *fliC* levels in WT, Δ*proQ*, and *proQ*-c strains. **(C)** Promoter activity of *fliC* in WT and Δ*proQ* strains, measured by a transcriptional reporter assay. **(D)** Stability of *fliC* in WT, Δ*proQ*, and *proQ*-c strains. Bacterial cultures were grown to an OD_600nm_ of 0.6, rifampicin was added to inhibit transcription, and qRT-PCR was performed at the indicated time points to assess the levels of *fliC* transcript. WT, EHEC O157:H7 wild-type strain; Δ*proQ*, *proQ* deletion strain; *proQ*-c, *proQ* complementation strain. The average values from three biological replicates with SD are shown. Statistical significance was assessed via one-way **(A)** or two-way ANOVA **(B)** analysis, or two-tailed Student’s t-test **(C)**. *, *P* ≤ 0.05; ***, *P* ≤ 0.001; ns, non-significant.

Besides LEE, flagellar genes also play critical roles in EHEC O157:H7 pathogenesis (Sun et al., 2022a). To test the importance of *proQ* on flagella production, we first compared the bacterial motility between WT and Δ*proQ*. As shown in **Figure 5A**, the Δ*proQ* strain exhibited severely impaired motility, with an approximately tenfold reduction in swimming radius after 10 hours on LB agar compared to WT. This defect was rescued by *proQ* complementation, suggesting that ProQ positively regulates motility of EHEC O157:H7.

The biosynthesis and assembly of a flagellum in *E. coli* is controlled by genes in three hierarchical classes (Chaban et al., 2015). The Class I gene *flhDC* encodes the master regulator, which activates Class II genes responsible for structural and assembly components of the hook-basal body, as well as the regulatory proteins FliA and FlgM. Class III genes (e.g., *fliC*, and *motAB*-*cheAW*) encode distal structural elements of the flagellum and proteins essential for flagellar rotation and chemotaxis. To determine which class of flagellar genes is involved in ProQ-mediated regulation of motility in EHEC O157:H7, we assessed the expression of *flhD*, *fliA*, *flgM*, and *fliC* following *proQ* deletion. Among these, only expression of *fliC* showed a significant reduction in the Δ*proQ* strain, while the expression of others remained unchanged (**Figure 5B**), suggesting that ProQ specifically modulates motility by regulating *fliC*. To further explore how *proQ* regulates *fliC*, we examined the effect of *proQ* deletion on transcription and stability of *fliC*. As indicated in **Figure 5C**, the *fliC* promoter activity was unaffected by *proQ* deletion. However, RNA decay assays revealed a remarkable decrease in *fliC* mRNA stability in the Δ*proQ* strain compared to WT and *proQ* complementation strains (**Table 1**; **Figure 5D**). These data strongly indicate that ProQ positively regulates the expression of *fliC* and bacterial motility of EHEC O157:H7 at the post-transcriptional level.

### ProQ promotes antibiotic persistence of EHEC

**Figure 6:**
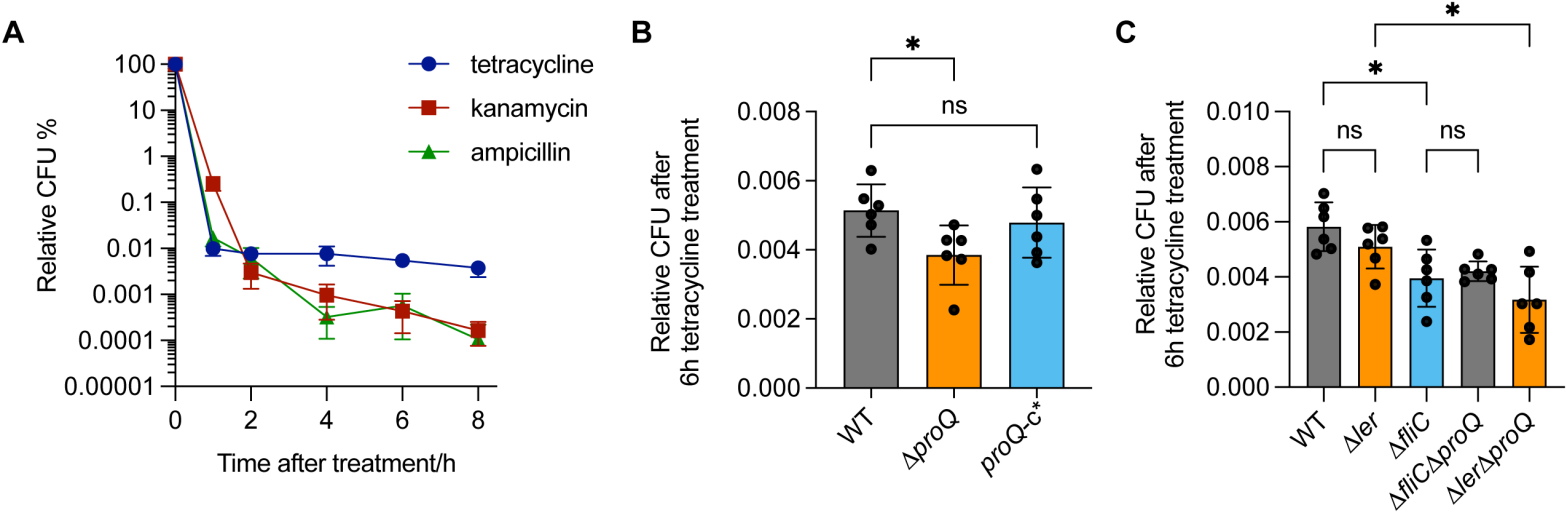
ProQ promotes antibiotic persistence of EHEC O157:H7 through *fliC*. **(A)** Antibiotic persistence of EHEC O157:H7. Exponential-phase cultures were treated with 50× MIC of tetracycline (75 µg/mL), kanamycin (15 µg/mL) and ampicillin (250 µg/mL). CFU counts were determined before and after treatments to calculate the relative surviving fraction at indicated time points. **(B)** Persistence of WT, Δ*proQ*, and *proQ*-c^*^ strains following tetracycline treatment. **(C)** Persistence of WT, Δ*proQ*, Δ*ler*, Δ*fliC*, Δ*proQ*Δ*ler*, and Δ*proQ*Δ*fliC* strains following tetracycline treatment. Strains were grown to an OD_600nm_ of 0.6, after which tetracycline was added to cell cultures at 50× MIC (75 μg/mL), followed by an incubation for 6 h. WT, EHEC O157:H7 wild-type strain; Δ*proQ*, *proQ* deletion strain; *proQ*-c^*^, *proQ* complementation strain constructed using the pWSK29 vector. The average values from six biological replicates with SD are shown. Statistical significance was assessed via one-way ANOVA analysis. *, *P* ≤ 0.05; ns, non-significance.

In addition to regulating virulence genes, ProQ has also been implicated in antibiotic persistence in several pathogenic bacteria (Rizvanovic et al., 2022; Cianciulli Sesso et al., 2024). For instance, in *Salmonella*, ProQ contributes to the formation of a subpopulation of growth-arrested cells capable of surviving high concentrations of various antibiotics (Rizvanovic et al., 2022). These findings prompted us to investigate whether EHEC O157:H7 also exhibits antibiotic persistence and whether ProQ plays a role in this process. We exposed the WT and Δ*proQ* strains to several antibiotics commonly used for EHEC treatment—including ampicillin, kanamycin, and tetracycline—at concentrations far above the MICs that are usually used to test bacterial antibiotic persistence (Balaban et al., 2019; Rizvanovic et al., 2022). These antibiotics have been shown to reduce EHEC infection without inducing Shiga toxin release or causing intestinal damage, making them promising options for effective treatment (Mühlen et al., 2020). After 1 hour of antibiotic exposure at 50× MICs, most EHEC O157:H7 cells were efficiently killed (**Figure 6A**). However, a biphasic pattern with a plateau of surviving persister was observed after 2 hours of tetracycline treatment and after 4 hours of ampicillin or kanamycin treatment (**Figure 6A**). To assess the role of ProQ in persister formation, we challenged the Δ*proQ* strain with tetracycline at 50× MIC for 6 hours and compared its survival to that of the WT and *proQ*-complemented strains. As shown in **Figure 6B**, deletion of *proQ* significantly reduced the persister level compared to the WT, and complementation restored persistence to the WT level. Notably, MIC testing confirmed that differences in survival were not due to altered antibiotic resistance (**Table 2**). These results demonstrate that ProQ is essential for persister formation in EHEC O157:H7 under antibiotic stress.

**Table 2:** Minimum inhibitory concentrations (MICs) determined for EHEC EDL933 strains.

Strain	MIC (µg/ml)		
	Tetracycline	Ampicillin	Kanamycin
WT	1.52 ± 0.08	5.04 ± 0.17	0.31 ± 0.02
Δ*proQ*	1.42 ± 0.25	5.23 ± 0.31	0.27 ± 0.04

The average values from three biological replicates with SD are shown.

It is worth noting that ProQ-dependent persister formation in *Salmonella* is associated with the activation of metabolically costly processes, such as virulence gene expression (Rizvanovic et al., 2022). In line with this, we found that deletion of *fliC* significantly reduced persister level after 6 hours of tetracycline treatment (**Figure 6C**), whereas deletion of *ler* had little effect. This suggests that flagellar synthesis, but not T3SS synthesis, contributes to antibiotic persistence in EHEC O157:H7. Furthermore, deletion of *proQ* did not further decrease persister levels in the Δ*fliC* background, whereas a similar effect was not observed in the Δ*ler* strain (**Figure 6C**). Collectively, these findings indicate that ProQ promotes antibiotic persistence in EHEC O157:H7 primarily through regulation of *fliC*, rather than *ler*.

### ProQ directly binds to virulence related genes through specific structure at the 3’ end

**Figure 7:**
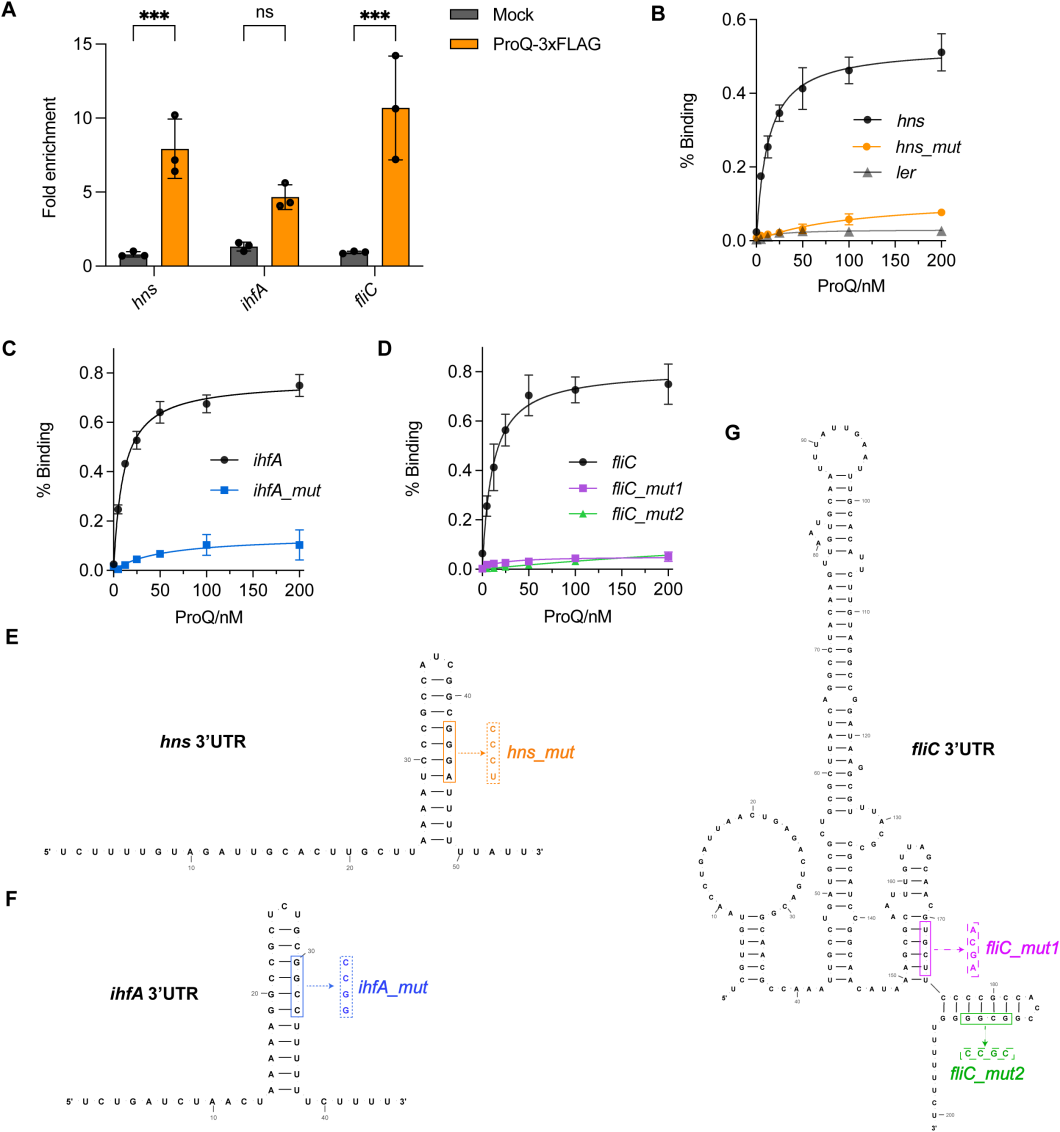
Direct binding of ProQ on *hns*, *ihfA* and *fliC*. **(A)** *In vivo* binding of ProQ to *hns*, *ihfA*, and *fliC*. RNA associated with ProQ-3×FLAG was purified by RNA co-immunoprecipitation (co-IP) from an EHEC O157:H7 strain expressing ProQ-3×FLAG, and enrichment of tested mRNAs was assessed by qRT-PCR. The average values from three biological replicates with SD are shown. ***, *P* ≤ 0.001. Statistical significance was assessed via two-way ANOVA analysis. **(B–D)** Assessment of binding between purified ProQ and *in vitro* transcribed 3’UTRs of *hns* **(B)**, *ihfA* **(C)** and *fliC* **(D)** as well as their corresponding variants carrying mutations that disrupt the hairpin structures. Full-length *ler* mRNA was tested and served as a negative control (shown in **B**). **(E–G)** Predicted secondary structures of the 3’UTRs of *hns* **(E)**, *ihfA* **(F)**, and *fliC* **(G)**. Mutations introduced to disrupt the hairpin structures for filter binding assays are indicated.

**Figure 8:**
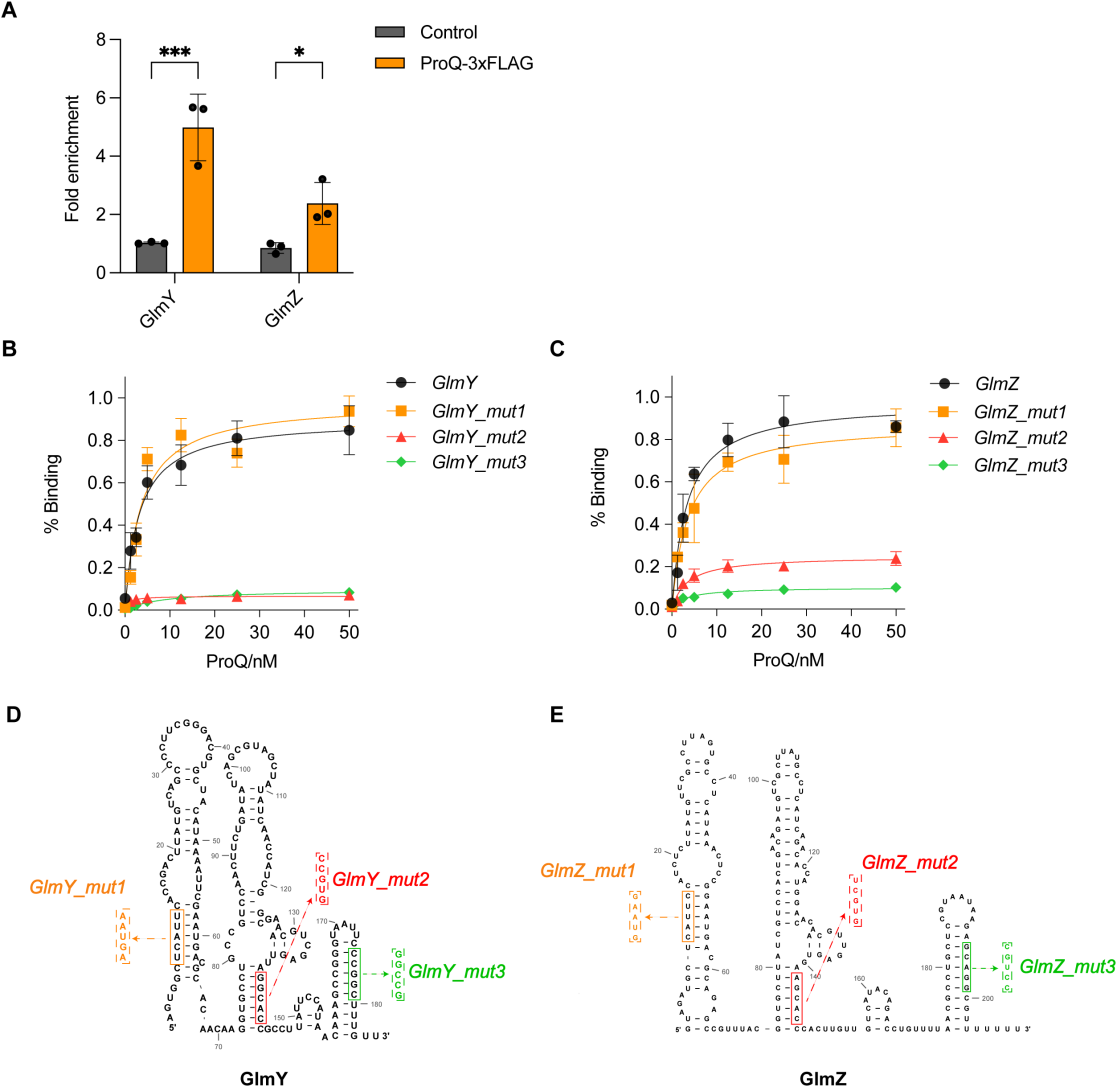
Direct binding of ProQ on GlmY and GlmZ. **(A)** *In vivo* binding of ProQ to GlmY and GlmZ. RNA associated with ProQ-3×FLAG was purified by RNA co-immunoprecipitation (co-IP) from an EHEC O157:H7 strain expressing ProQ-3×FLAG, and enrichment of tested sRNAs was assessed by qRT-PCR. The average values from three biological replicates with SD are shown. *, *P* ≤ 0.05; ***, *P* ≤ 0.001. Statistical significance was assessed via two-way ANOVA analysis. **(B, C)**. Assessment of binding between purified ProQ and *in vitro* transcribed GlmY **(B)** or GlmZ **(C)**, along with variants carrying mutations in each of the three hairpin structures. **(D, E)**. Predicted secondary structures of full-length GlmY **(D)** and GlmZ **(E)**. Mutations introduced to disrupt the hairpin structures for filter binding assays are indicated.

While we have demonstrated the regulatory roles of ProQ in several genes and sRNAs important for EHEC O157:H7 virulence, it remained unclear whether and how ProQ directly binds these RNAs. To investigate this, we first performed RNA co-immunoprecipitation (co-IP) experiments and assessed the enrichment of *hns*, *ihfA*, and *fliC* mRNAs, as well as the sRNAs GlmY and GlmZ. All tested RNAs were enriched in the ProQ-coIP samples (**Figures 7A**, **8A**), indicating that ProQ binds these RNAs *in vivo*. To further confirm direct binding, we conducted filter binding assays using purified ProQ protein and radiolabeled *hns*, *ihfA*, and *fliC* mRNAs. As shown in **Figures 7B–D**, ProQ bound directly to all three mRNAs. In contrast, no binding was detected for *ler* (**Figure 7B**). Importantly, hairpin structures were found in the 3’ UTRs of *hns*, *ihfA*, and *fliC* (**Figure 7E**). Given that ProQ typically recognizes RNA substrates through structured elements at the 3’ end, we generated mutated versions of these mRNAs (*hns*/*ihfA*/*fliC* mut) in which the hairpin structures were disrupted (**Figure 7E**). Binding assays revealed that these mutations severely diminished or abolished ProQ binding (**Figures 7B–D**), demonstrating that ProQ directly interacts with *hns*, *ihfA*, and *fliC* through these specific structures. These findings are consistent with previous studies showing that ProQ preferentially binds RNAs with distinct secondary structures (Holmqvist et al., 2018; Mamonska et al., 2025).

We also confirmed the *in vitro* binding between ProQ protein with GlmY and GlmZ (**Figures 8B, C**), with similar binding affinities observed for these two sRNAs. Considering that GlmY and GlmZ were previously reported to contain three prominent hairpin structures (Urban and Vogel, 2008), we tested variants of GlmY and GlmZ carrying mutations that disrupted each individual hairpin to identify which hairpins are critical for ProQ binding (**Figure 8D**). Interestingly, disruption of the 5’ hairpin did not significantly affect ProQ binding affinity for both sRNAs, whereas mutations in the middle and 3’ hairpins substantially impaired ProQ binding (**Figures 8B, C**). These results suggest that the central and 3’ hairpins are essential for the interaction between ProQ and GlmY/GlmZ.

### Effect of ProQ on LEE and *fliC* expression is conserved in other EHEC and EPEC strains

**Figure 9:**
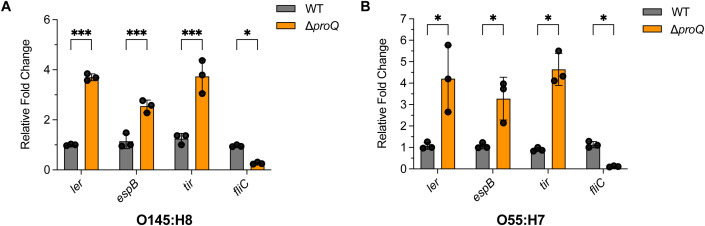
The regulation of ProQ on LEE and *fliC* genes is conserved in EHEC O145:H8 and EPEC O55:H7 strains. qRT-PCR analysis of *ler*, *espB*, *tir*, and *fliC* levels in WT, Δ*proQ*, and *proQ*-c strains of EHEC O145:H8 **(A)** or EPEC O55:H7 **(B)**. WT, Δ*proQ*, and *proQ*-c strains. WT, wild-type strain; Δ*proQ*, *proQ* deletion strain; *proQ*-c, *proQ* complementation strain. The average values from three biological replicates with SD are shown. Statistical significance was assessed via two-way ANOVA analysis. *, *P* ≤ 0.05; ***, *P* ≤ 0.001.

ProQ is a conserved protein among many bacterial pathogens, suggesting that it mediates similar post-transcriptional regulatory mechanisms across different species. To further test this, we examined the influence of *proQ* deletion on LEE and *fliC* expression in EHEC O145:H28 (a second prevalent EHEC species) and EPEC O55:H7 (the ancestor of EHEC O157H7). qRT-PCR analysis revealed that deletion of *proQ* orthologous genes significantly elevated expression of *ler*, *espB* and *tir*, but reduced level of *fliC* gene in both strains (**Figure 9**). Similar regulatory effects of *proQ* were also previously reported in another EPEC serotype O127:H6 (Mihaita et al., 2025). These results suggest that the regulatory roles of ProQ on LEE and *fliC* gene expression is conserved in a range of EHEC and EPEC strains.

## Discussion

Coordinating gene expression is crucial for bacterial pathogens (e.g., EHEC) to survive hostile conditions and to evade immune defenses, enabling successful colonization of the host intestine (Connolly et al., 2015; Woodward et al., 2019; Wale et al., 2021). As a result, virulence-specific regulators are often tightly controlled at both the transcriptional and post-transcriptional levels through intricate regulatory networks that integrate environmental signals. In this study, we revealed important roles of the RNA chaperone ProQ on EHEC virulence gene expression. We demonstrated that ProQ negatively affects LEE expression through post-transcriptional regulation of several factors controlling LEE, including *hns*, *ihfA*, and the sRNAs GlmY and GlmZ. ProQ also positively regulates the bacterial motility through stabilizing *fliC*. We further showed that the regulatory effect of ProQ on these virulence genes is mediated by direct binding on the hairpin structures at the 3’ end. Consequently, deletion of *proQ* caused impaired colonization, stress adaption and antibiotic persistence of EHEC.

Our data showed that disruption of *proQ* significantly promotes the adherence of EHEC to human colorectal epithelial cells (**Figure 1F**), likely due to upregulation of the LEE pathogenicity island (**Figure 2A**). ProQ indirectly regulates *ler*, the master LEE activator, as no binding was detected between ProQ and *ler* mRNA *in vivo* or *in vitro* (**Figures 2D**, **7B**). Given the hierarchical nature of LEE regulation, modulation of *ler* is likely a central mechanism through which ProQ controls LEE expression. Deletion of *proQ* increased *ler* promoter activity and stabilized the *ler* mRNA (**Figures 2B, C**), suggesting that this regulation occurs at both transcriptional and post-transcriptional levels. Our data imply that H-NS (encoded by *hns*) and IHF (encoded by *ihfA* and *ihfB*) may mediate the transcriptional regulatory effect of *proQ* on LEE, as *proQ* stabilizes the transcripts of *hns* and *ihfA*. H-NS represses LEE under non-permissive conditions (Levine et al., 2014), while IHF activates *ler* by directly binding to a DNA region upstream of the *ler* promoter (Friedberg et al., 1999). Interestingly, ProQ promotes the stability of both *hns* and *ihfA* mRNAs (**Figures 3B, C**), despite their opposing effects on LEE expression. The reason for this remains unclear. We also examined the effect of *proQ* deletion on *ihfB*, which encodes the other subunit of the IHF heterodimer. No difference in *ihfB* levels was observed between WT and Δ*proQ* strains (**Supplementary Figure S4**), suggesting that ProQ specifically affects *ihfA*, but not *ihfB*. Since we only examined three known LEE regulators in this study, it is likely that ProQ influences additional, uncharacterized factors, which may make HNS the dominant regulator of LEE under the tested conditions.

The post-transcriptional regulatory effect of ProQ on LEE is likely mediated through the sRNAs GlmY and GlmZ (**Figures 4A–C**). These sRNAs are known to impact genes associated with amino sugar metabolism in *E. coli* and related bacteria (Urban and Vogel, 2008). Specifically, GlmZ directly interacts with the *glmS* mRNA, which encodes glucosamine-6-phosphate synthase, and activates its translation. GlmY does not directly bind *glmS* mRNA, but stabilizes GlmZ by sequestering RapZ, a protein that promotes GlmZ degradation (Khan et al., 2020). In EHEC, GlmY and GlmZ retain their core regulatory roles in *glmS* expression but also contribute to the broader regulatory networks linked to virulence (Gruber and Sperandio, 2014, 2015). Deletion of GlmY and GlmZ altered expression of several LEE, curli adhesin, tryptophan metabolism genes and non-LEE-encoded effectors (Gruber and Sperandio, 2015), which are also affected by the deletion of *proQ* (**Figure 4D**). Importantly, GlmZ directly binds and destabilizes the 3’ region of LEE4 and LEE5 operons (Gruber and Sperandio, 2014). In contrast, overexpression of both GlmY and GlmZ did not affect *ler* mRNA levels (Gruber and Sperandio, 2014). Given the negative regulation of *proQ* on *ler* at the posttranscriptional level (**Figure 2**), other sRNAs are likely involved in this process. Thus, ProQ may orchestrate a multi-layered regulatory network involving both transcriptional regulators and multiple sRNAs to fine-tune LEE expression. However, the specific additional sRNAs in EHEC contributing to this regulation remain unidentified. Although five known LEE-regulating sRNAs were examined, none displayed altered expression upon *proQ* deletion (**Figure 4A**). Further investigation will be required to resolve this question.

In addition to LEE, ProQ also plays a significant role in regulating motility of EHEC (**Figure 5A**). ProQ promotes the stability of *fliC* mRNA which encodes flagellin, the major structural component of the bacterial flagellum (**Figure 5B**). However, ProQ has no effect in *fliC* promoter activity and other master transcriptional regulator of flagella biogenesis, such as *flhD* and *flgM* (**Figures 5B–D**), suggesting that *proQ* only regulates *fliC* at the post-transcriptional level. Notably, *proQ* has distinct regulatory effects on flagella and LEE regulation, in line with the inverse relationship between these two virulence factors during EHEC infection. Early in infection, EHEC prioritizes motility to reach and colonize the intestinal epithelium, leading to high expression of flagellar genes. However, once the bacterium approaches the host surface, motility is downregulated, and LEE expression is upregulated (Connolly et al., 2015). This reciprocal regulation ensures an efficient transition from a motile, exploratory state to a sessile, virulence-focused state, minimizing detection by the host immune system while maximizing colonization efficiency. ProQ appears to help coordinate this switch, reflecting a sophisticated regulatory network that balances movement and adherence during different stages of EHEC infection.

Importantly, ProQ directly binds to the hairpin structures located at the 3’ end of *hns*, *ihfA*, *fliC*, GlmY, and GlmZ (**Figures 7**, **8**). The 3’ end structures of these genes are conserved at the structural level across *Salmonella*, enteropathogenic *E. coli* O55:H7 (EPEC), and *E. coli* K-12, despite low sequence homology (**Supplementary Figure S5**). This observation aligns with the known binding preference of ProQ for structured RNA elements, particularly stem-loops or terminator-like hairpins often found at the 3’ ends of transcripts, rather than specific sequence motifs (Holmqvist et al., 2018; Mamonska et al., 2025). Intriguingly, in addition to their 3’ terminal hairpins, GlmY and GlmZ harbor internal hairpin structures that also interact with ProQ (**Figures 8B, C**), indicating that ProQ is capable of engaging with multiple structural elements within a single RNA molecule. Of particular interest, the internal hairpin of GlmZ also interact with the 3’ region of the LEE4–5 transcripts (Gruber and Sperandio, 2014), implying that ProQ binding may influence GlmZ’s regulatory function on its RNA targets, which mirrors the function of ProQ in RaiZ-*hupA* regulation (Smirnov et al., 2017). Together, these findings highlight the structural basis of ProQ-RNA recognition and its potential to orchestrate complex regulatory interactions involved in EHEC virulence.

Notably, both GlmY and GlmZ are also capable of binding to the major sRNA chaperone Hfq in *E. coli* (Melamed et al., 2020). Hfq has been shown to play a key role in regulating virulence genes in various EHEC strains (Shakhnovich et al., 2009; Sudo et al., 2022). Although Hfq represses the LEE regulator *ler* by directly binding its mRNA in an sRNA-independent manner (Shakhnovich et al., 2009; Sudo et al., 2022), it remains possible that other sRNAs—such as GlmY and GlmZ—may modulate additional LEE genes with Hfq’s assistance. Interestingly, we did not detect direct binding of ProQ to *ler* (**Figure 2D**), suggesting that ProQ regulates LEE through a different mechanism than Hfq. This further implies that ProQ likely acts through sRNAs, such as GlmY and GlmZ, to influence LEE expression. In the non-pathogenic *E. coli* K-12, Hfq and ProQ have been shown to function in overlapping, complementary, or even competing ways in post-transcriptional sRNA-mediated regulation (Melamed et al., 2020). However, their interplay in the context of EHEC virulence remains poorly understood. Elucidating how Hfq and ProQ coordinate or compete in controlling virulence gene expression will be crucial for unraveling the complexity of RNA-based regulation in this important pathogen.

Our work also revealed similar effects of ProQ on LEE and *fliC* expression across multiple EHEC and EPEC strains (**Figure 9**), consistent with observations from a recent study of ProQ regulation in the EPEC O127:H6 strain (Mihaita et al., 2025). Both studies found that *proQ* deletion similarly affects LEE expression, biofilm formation, and motility in EHEC and EPEC. However, the underlying regulatory mechanisms appear to differ between these species. For example, in EPEC, the effect of ProQ on *ler* expression is modulated by the plasmid-encoded regulator PerC, which is absent in EHEC. While *ler* promoter activity increases upon *proQ* deletion in both EHEC and EPEC, this effect in EHEC is likely mediated by reduced expression of *hns*, a known LEE repressor. Additionally, ProQ contributes to biofilm formation in EHEC by activating the master regulator CsgD, potentially through the small RNAs GlmY and GlmZ. In contrast, in EPEC, *csgD* transcript levels are not significantly altered in the Δ*proQ* mutant, although ProQ still impacts biofilm formation and expression of the CsgD-regulated *csgBAC* operon, indicating that ProQ might regulate biofilm-related genes independent of CsgD in EPEC. These findings suggest that EHEC and EPEC employ distinct regulatory pathways to achieve similar outcomes in conserved virulence traits. Notably, the mechanism by which ProQ regulates motility appears to be conserved, as it operates primarily through modulation of *fliC* expression in both pathogens. Future comparative studies between EHEC, EPEC, and other related pathogens will be essential to further dissect the species-specific and conserved roles of ProQ in virulence regulation.

The low infectious dose of EHEC, and its ability to survive in diverse environmental conditions, makes it a particularly difficult pathogen to control. While antibiotics like ampicillin, tetracycline, and kanamycin can reduce EHEC without inducing host cell damage and Shiga toxin, here we provide the first evidence that prolonged treatment using them can lead to persister formation in EHEC (**Figure 6A**). Notably, this persistence phenotype depends on the expression of *proQ* and *fliC*, as well as *proQ*-mediated regulation of *fliC* (**Figures 6B, C**). In contrast, *ler* and its regulation by *proQ* appear to play a minimal role in persister formation (**Figure 6C**). This contrasts with *Salmonella*, where both T3SS and flagellar genes contribute to antibiotic persistence (Rizvanovic et al., 2022). The discrepancy may stem from the distinct pathogenic strategies of these organisms—EHEC being a non-invasive pathogen, whereas *Salmonella* invades and replicates within host cells to cause systemic infection. Additionally, deletion of *proQ* remarkedly impaired the survival of EHEC O157:H7 in the murine gastrointestinal tract and under host-derived stress conditions (**Figures 1A–D**). These findings indicate that ProQ can serve as a key regulator of both persistence and virulence in EHEC—and a promising target for future anti-EHEC strategies.

## Materials and methods

### Bacterial and cell growth condition

The bacterial strains (see a complete list of strains in **Supplementary Table S1**) were grown in standard Luria-Bertani (LB) media with shaking at 200 rpm at 37°C. When required, antibiotics were added (chloramphenicol at 35 μg/mL or kanamycin at 50 μg/mL). Caco-2 cells were purchased from ATCC and maintained at 37°C with 5% CO_2_ in Dulbecco’s modified Eagle medium (DMEM; ThermoFisher) supplemented with 10% heat-inactivated fetal calf serum (GE Healthcare) and 10,000 U/mL of Penicillin-Streptomycin (ThermoFisher) and G418 (Sigma) at 500 μg/mL.

### Construction of strains and plasmids

The isogenic mutants lacking *proQ* in EHEC or EPEC strains were created using the λ red recombination technique, using pKD4 to create the deletion PCR products and pCP20 to remove the antibiotic cassette (Datsenko and Wanner, 2000). The mutant was confirmed by colony PCR and Sanger sequencing. The chromosomally tagged ProQ strain was constructed by replacing the stop codon with a 3×FLAG sequence using the same strategy as for gene deletion. The complementation plasmid expressing *proQ* was engineered by cloning the coding sequence of *proQ* and its native promoter (100 nt upstream of the transcriptional start site) between the XbaI/SphI restriction sites on the low copy plasmid pACYC184, or the SacII/BamHI sites on pWSK29. The constructed plasmids were transformed into the Δ*proQ* to construct the complementation strains. To construct the plasmid for ProQ and EspB purification, the coding sequence of *proQ* or *espB* was amplified by PCR and cloned in between the SacII/BamHI restriction sites on the pET28a plasmid. The recombined plasmids were transformed into the BL21 (DE3) strain. The transcriptional reporter fusions were constructed by inserting sequences corresponding to -150 – +10 nt relative to the transcriptional start site of each targeted gene (*hns*, *ihfA*, and *fliC*) between into XhoI/BamHI digested pMS402 plasmid. For *ler-lux* construction, we used the sequence -300 to +10 bp with respect to the transcription start site of the proximal promoter.

All plasmids and primers used in this study are listed in **Supplementary Table S2** and **Supplementary Table S3**, respectively.

### RNA isolation

Total RNA was extracted from bacterial cultures growing at OD_600nm_ of 0.6 in LB media. Briefly, 10 OD of bacterial cell pellets were collected and resuspended in 1 mL TRIzol Reagent (ThermoFisher). Cells were mechanically lysed by beadbeating on ice (using 5 cycles of 10 s on and 20 s off). Supernatant was collected by centrifugation for 10 min at 8,000g, 4°C. 300 μL of phenol: chloroform:isoamyl alcohol (25:24:1, pH4.5, Roth) was then added, mixed with the cell lysates, and incubated for 3 min at room temperature, followed by centrifugation for 15 min at 13,000g, 4°C. The upper aqueous phase was removed and transferred to a new tube for another round of extraction using 300 μL chloroform (Sigma-Aldrich). RNA was mixed with 2.5 volume of ice-cold ethanol and 0.3 M sodium acetate, followed by the incubation at -20°C for overnight. RNA pellets were collected by centrifugation for 10 min at 13,000g, 4°C, washed twice with ice-cold 75% ethanol and resuspended in 50 μL of nuclease-free water (ThermoFisher). RNA was then treated with DNase I (ThermoFisher) (0.25 U per 1 µg of RNA) for 45 min at 37°C and further purified using the same strategy as described above. The integrity of purified RNA was evaluated by electrophoresis. The concentration of RNA samples was determined by Nanodrop (ThermoFisher).

### Quantitative real-time quantitative RT-PCR

RT-qPCR was performed using the Luna one step RT-qPCR kit (New England Biolabs) and the Applied Biosystems ViiA 7 instruments with QuantStudio Real-Time software v1.3 (Applied Biosystems) according to the manufacturer’s instructions. For qRT-PCR analysis of sRNAs with short length (DicF, GlmY and GlmZ), primers carrying overhang sequences were used, following a protocol published before (Sharbati-Tehrani et al., 2008). Data were normalized to the expression levels of the 16S rRNA gene (*rrsH*). Fold changes in expression were determined using the 2^-ΔΔCT^ method (Schmittgen and Livak, 2008). Three biological replicates were analyzed for each experiment. Primer sequences used for qRT-PCR are summarized in **Supplementary Table S2**.

### Bacterial adherence assay

Bacterial adherence assay was performed as described previously (Liu et al., 2020). Briefly, bacteria were grown in LB media with an OD_600nm_ of 0.6, collected by centrifugation at 4,000g for 3 min. The cells were washed with and resuspended in DMEM medium and added to Caco-2 epithelial cells at a multiplicity of infection (MOI) of 100. At 3 h post-infection, the Caco-2 cells were washed three times with sterile 1× PBS to remove non-attached bacteria and lysed with 1 mL of 0.1% Triton X-100 for 5 min. The cell lysates were serially diluted with sterile 1× PBS and plated on LB agar plates. After incubation at 37°C overnight, the bacterial colonies were counted. The adhesion efficiency of different strains was compared by calculating the number of adhered bacteria per mL.

### Transcriptional reporter assay

The plasmids carrying transcriptional fusions were transformed into the WT or Δ*proQ* strains. The resulting strains were then grown at 37°C in LB media to an OD_600nm_ of 0.6. Cells were washed with and resuspended in sterile 1× PBS. 200 µl of each diluted cell culture was added to 96-well plates (Corning), and the *lux* signal intensity was recorded using the Infinite M200 PRO plate reader (Tecan). The OD_600nm_ was also measured in parallel for normalization.

### RNA half-life determination

Bacterial cultures were grown to an OD_600nm_ of 0.6, and 250 µg/ml rifampicin was added. Cells were incubated at room temperature and 4 OD samples were collected at different time intervals post-treatment. Each sample was immediately mixed with 1/5 volume of 95% ethanol and 5% phenol and frozen in liquid nitrogen. Total RNA extraction and qRT-PCR analysis were carried out as described above.

### Enzyme-linked immunosorbent assay

ELISA assays were performed to evaluate the secretion of EspB in EHEC O157:H7 as described previously (Carlson-Banning and Sperandio, 2016). Bacterial cultures were grown in LB medium at 37°C to an OD_600nm_ of 0.6. Cell growth was then quenched with STOP solution (0.92 M sodium azide and 100 µl of Sigma protease inhibitor cocktail in sterile 1× PBS). The reaction mixtures were diluted 1:2 with sterile 1× PBS and incubated in Dynatech Laboratories Microtiter ELISA plates. Wells were blocked with 5% milk in PBST (1× PBS with 0.1% Tween 20), and samples were washed with PBST before incubation with an anti-EspB primary antibody (1:1,000; ANTIBODIES-ONLINE), followed by a goat anti-rabbit HRP-conjugated secondary antibody (1:1,000; ThermoFisher). Plates were developed using a 3,3’,5,5’-Tetramethylbenzidine substrate (Sigma-Aldrich) and the reaction was stopped with 2N HCl. Absorbance was measured at 450 nm using a Tecan plate reader. Absolute EspB concentrations were determined from standard curves generated by titrating purified EspB protein of known concentration, buffer-exchanged into 1× PBS. Two independent experiments were performed, each with three biological replicates.

### RNA co-immunoprecipitation

To purify ProQ-associated RNAs *in vivo*, RNA co-immunoprecipitation (RNA co-IP) assays were performed as previously described (Smirnov et al., 2016). An EHEC O157:H7 strain carrying chromosomally 3xFLAG-tagged ProQ were grown at 37°C in LB media to an OD_600nm_ of 0.6. 50 OD of bacteria were harvested by centrifugation and lysed as described in the “RNA Isolation” section. The cell lysates were split into two: one half incubated with 40 µl of monoclonal anti-FLAG M2 antibody (Sigma-Aldrich) for 30 min at 4°C (as the co-IP sample), while the other was left untreated with antibody and served as the mock co-IP control. 75 µl of prewashed Protein A Sepharose (Sigma-Aldrich) was then added and incubated for another 30 min at 4°C. Afterwards, Sepharose were washed extensively with the lysis buffer and resuspended in the lysis buffer. An equal volume of phenol:chloroform:isoamyl alcohol (25:24:1, pH4.5, Roth) was added for RNA exaction. RNA samples were further purified through two rounds of chloroform extractions and precipitated with ice-cold ethanol and 0.3 M sodium acetate.

To assess if one RNA is co-purified with ProQ-3xFLAG (indicating a direct binding), the RNA samples obtained from RNA co-IP experiments were subjected to qRT-PCR using specific primers targeting genes of interest. The enrichment of target RNAs in the co-IP or mock-coIP experiments was calculated as the ratio of abundance between the FLAG-tagged strains (FLAG) and the untagged control strain (WT). Each experiment was performed in three biological replicates.

### Filter binding assay

The ProQ purification was carried out using a HisTrap crude column (GE Healthcare) on a FPLC system (AKTA pure) as described before (Stein et al., 2020). RNA was transcribed using the MEGAshortscript T7 Transcription Kit (ThermoFisher) following the manufacturer’s instructions. The DNA templates used in *in vitro* transcription reactions (sequences shown in **Supplementary Table S3**) were synthesized by IDT. P^32^ labelling and purification of the tested RNAs were performed as described before (Han et al., 2022). Filter binding assay was performed by incubating purified ProQ proteins at various concentrations (0, 5, 12.5, 25, 50, 100, and 200 nM for mRNA binding, and 0, 1.25, 2.5, 5, 12.5, 25, and 50 nM for sRNA binding) with different radiolabeled RNA targets, followed by the protocol described before (Rio, 2012). Each filter binding assay was conducted in two (for GlmY/GlmZ and *ler*) or three (for *hns*, *ihfA* and *fliC*) replicates.

### Growth curve measurement and stress tolerance assays

To test if *proQ* affects the growth of EHEC O157:H7 under normal growth condition, bacterial overnight cultures were diluted into fresh LB media to achieve an OD_600nm_ of 0.03. The cultures were then grown at 37°C with shaking at 200 rpm. The OD_600nm_ values was measured every hour for 14 h using a spectrophotometer (Tecan). Each strain was analyzed in triplicate and the average OD_600nm_ values were used to plot growth curves. To test the effect of *proQ* on resistance of EHEC O157:H7 under acidic condition, pre-cultures of EHEC O157:H7 WT, *proQ* deletion and *proQ* complementation strains grown in LB media at 37°C (at an OD_600nm_ of 0.6) were diluted 1:100 in fresh LB with a pH adjusted to 2.5 with HCl. The cells were then incubated at 37°C with shaking at 200 rpm for 2 h. Resistance to bile salts, HD-5 and oxidative stress was determined by incubating bacterial cells grown in LB at an OD_600nm_ of 0.6 with 1% bile salts or 25 mM HD-5 at 37°C for 1 h, or with 5 mM H_2_O_2_ for 30 min at 4°C in the dark, respectively. Final bacterial cultures were serially diluted and plated onto LB plates for overnight incubation to determine viability. The survival rate of the cells was determined by comparing the viable counts of the challenged cells to those of the initial inoculum. For all these assays, two independent experiments were carried out with three biological replicates each.

### Motility assay

Strains of WT, *proQ* deletion and *proQ* complementation were cultured at 37°C in LB media to an OD_600nm_ of 0.6. 1 µl of cell cultures were spotted on the center of freshly made 0.3% soft agar plates. After incubation for 10 h at 37°C, diameter of the swimming zone for each strain was measured. All strains were tested in biological triplicate.

### Biofilm formation

Overnight cultures were diluted to an OD_600nm_ of 0.01 in fresh LB media in flat-bottom 96-well microtiter plates (Corning) and grown at 30°C for 48 h under static condition to enable biofilm formation. Wells were washed and cells stained with 0.1% crystal violet (Millipore Sigma) for 15 min. Crystal violet was then removed from the wells, followed by extensive washing with distill water. The remaining dye in the wells was dissolved in 200 μl of ethanol-acetone (4:1) solution and quantified by measuring the OD_590nm_ values. All strains were tested in biological triplicates, and the experiments were independently repeated twice.

### RNA structure prediction

RNA structures were predicted by RNAfold (http://rna.tbi.univie.ac.at/cgi-bin/RNAWebSuite/RNAfold.cgi) (Garcia-Martin et al., 2013) and ViennaRNA (http://rna.tbi.univie.ac.at/forna/) (Gruber et al., 2015), and drawn using R2DT (https://r2dt.bio/) (McCann et al., 2025) and RNACanvas (https://rna2drawer.app/) (Johnson and Simon, 2023).

### Antibiotic persistence assay

WT or deletion strains were grown in LB media at 37°C until an OD_600nm_ reached 0.6. Tetracycline (75 μg/mL), kanamycin (15 μg/mL), or ampicillin (250 μg/mL) were then added. After 1, 2, 4, 6, or 8 h, cells were washed three times and diluted in sterile 1× PBS and serially diluted for plating on agar plates. CFU counts of antibiotic treated and untreated cells were determined after overnight incubation at 37°C. The experiments were performed in six biological replicates.

### Statistical analysis

Unless otherwise indicated, all data are expressed as the mean ± standard deviation (SD) from three independent biological replicates. Statistical analyses were assessed using two-tailed Student’s t-test, one-way ANOVA or two-way ANOVA analysis, or two-sided Mann-Whitney rank-sum test. Differences were considered statistically significant when *P* ≤ 0.05 (^*^), while *P* values of ≤ 0.01 and ≤ 0.001 were considered highly and extremely significant (^**^ and ^***^), respectively.

## Data Availability

The datasets presented in this study can be found in online repositories. The names of the repository/repositories and accession number(s) can be found in the article/**Supplementary Material**.
