# Supplementary material for: The RNA-binding protein ProQ directly binds and regulates virulence genes in enterohemorrhagic Escherichia coli O157:H7
Source: Front Cell Infect Microbiol. 2025 Aug 29;15:1627518. doi: 10.3389/fcimb.2025.1627518 (PMC12426089; doi:10.3389/fcimb.2025.1627518)
Supplement: Supplementary file 1 [file Table1.docx]

Supporting Information for:

**The RNA-Binding Protein ProQ Directly Binds and Regulates Virulence Genes in Enterohemorrhagic *Escherichia coli* O157:H7**

Ye Qian^1,#^, Chenguang Zheng^2,#,*^, Runhua Han^3,*^

^1^School of Basic Medical Sciences, North China University of Science and Technology, Tangshan, China, 063210

^2^School of Public Health, North China University of Science and Technology, Tangshan, China, 063210

^3^Department of Chemistry, University of Manitoba, Winnipeg, MB, Canada, R3T 2N2

*****Correspondence: runhuahan@gmail.com, cgzheng@ncst.edu.cn

**^#^**These authors contributed equally to this work

**Supplementary Figures**

**
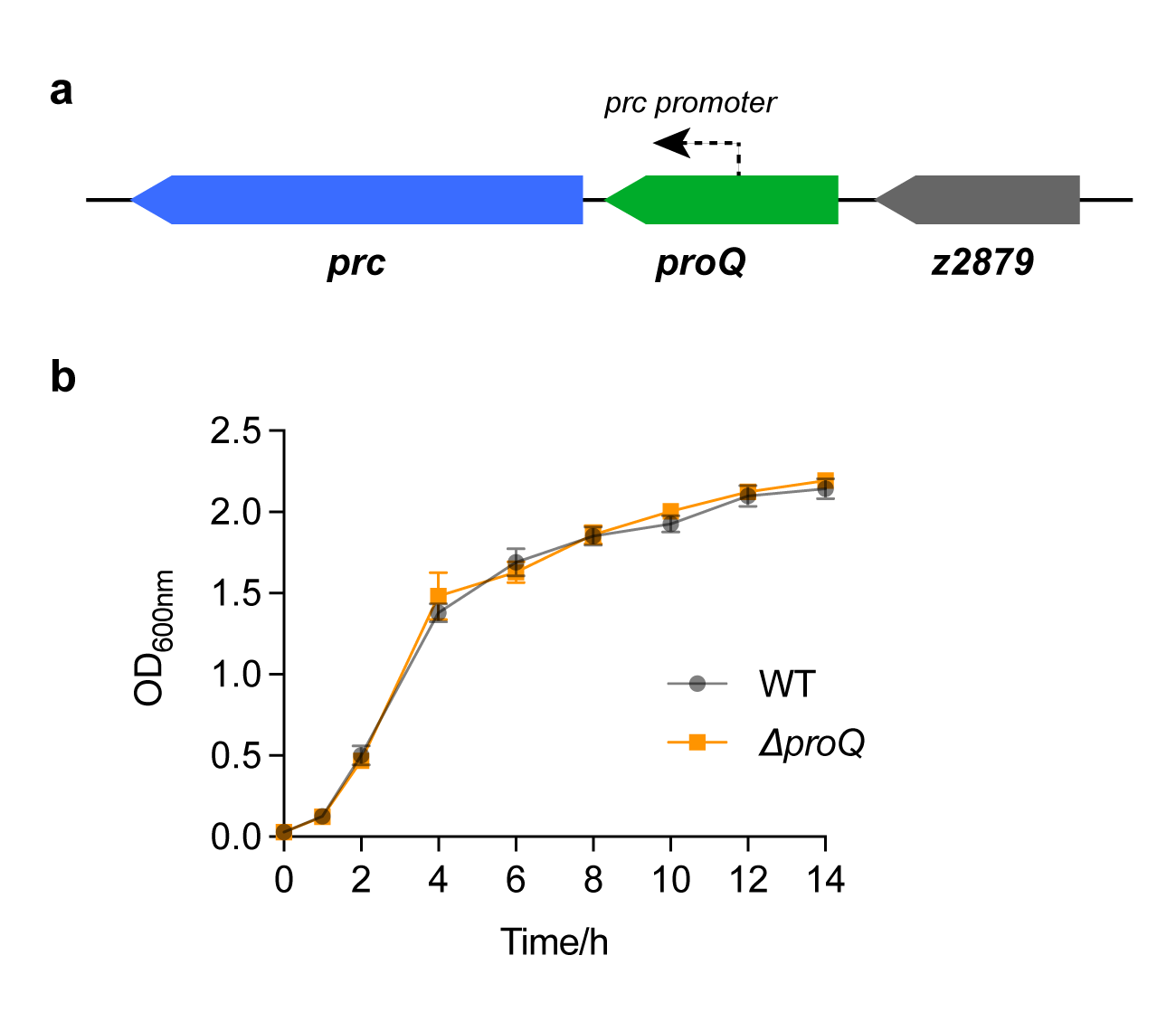
**

**Figure S1**. **The gene *proQ* and its effect on EHEC O157: H7 growth.** (A) Genomic localization of *proQ* in the EHEC O157:H7 EDL933 strain, with the promoter start site of *prc* highlighted. (B) Aerobic growth of WT and ∆*proQ* strains in LB medium at 37°C. WT, EHEC O157:H7 wild-type strain; ∆*proQ*, *proQ* deletion strain. The average values from three replicates with SD are shown.

**
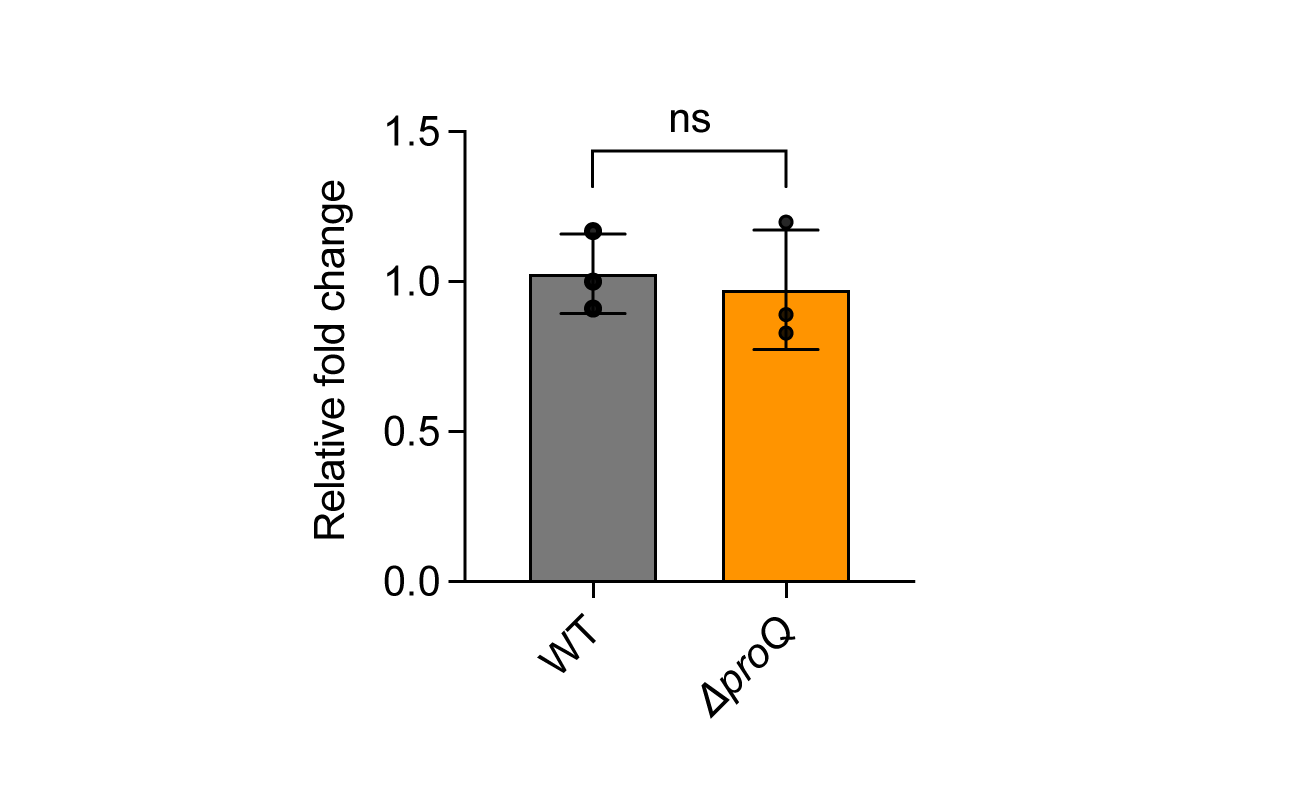
**

**Figure S2. Evaluation of effects of *proQ* deletion in EHEC O157:H7 on *prc* expression.** qRT-PCR analysis of *prc* levels in EHEC O157:H7 WT and ∆*proQ* strains. WT, EHEC O157:H7 wild-type strain; ∆*proQ*, *proQ* deletion strain. The average values from three biological replicates with SD are shown. Statistical significance was assessed via two-tailed Student’s t-test. ns, non-significant.

**
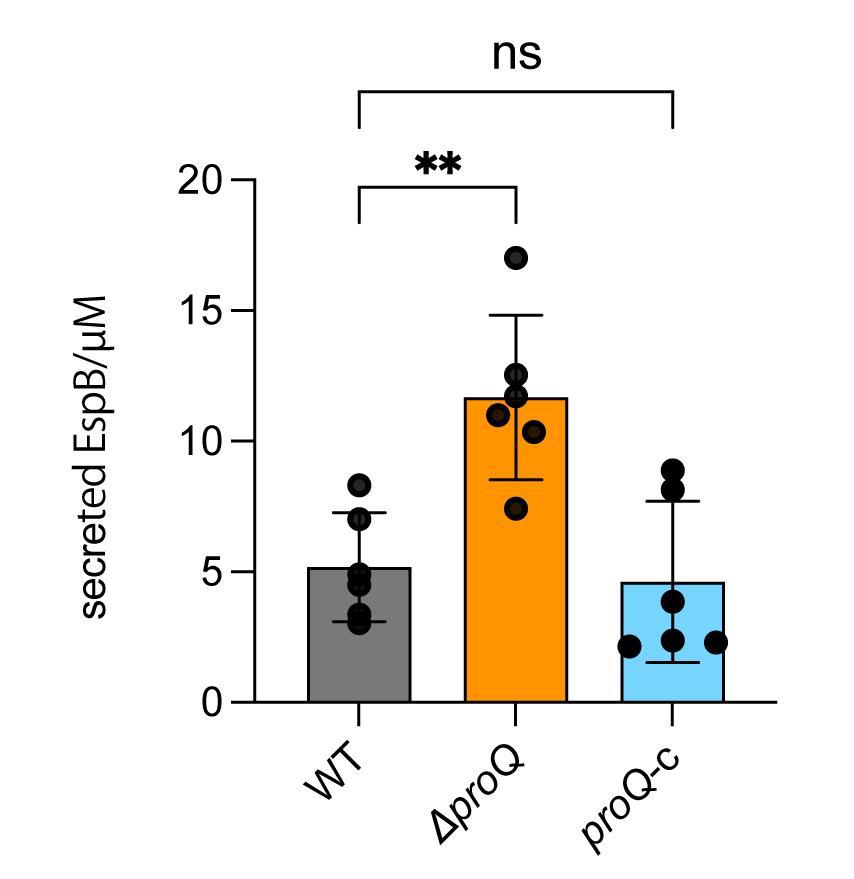
**

**Figure S3**. **EspB protein levels from EHEC O157:H7 cultures measured by ELISA.** Average values from six biological replicates with SD are shown. WT, EHEC O157:H7 wild-type strain; ∆*proQ*, *proQ* deletion strain; *proQ*-c^*^, *proQ* complementation strain constructed using the pWSK29 vector. Statistical significance was assessed via one-way ANOVA analysis. ^**^, *P* ≤ 0.01; ns, non-significant.

**
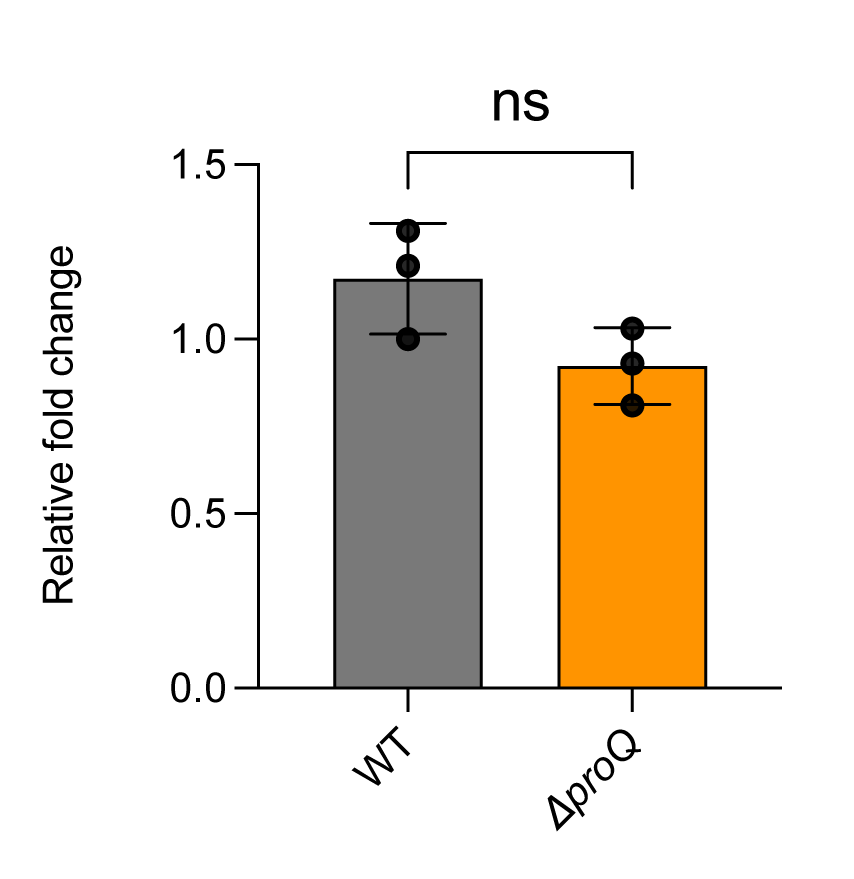
**

**Figure S4. Expression of *ihfB* in EHEC O157:H7 WT and ∆*proQ* strains.** Average values from three biological replicates with SD are shown. WT, EHEC O157:H7 wild-type strain; ∆*proQ*, *proQ* deletion strain. Statistical significance was assessed via two-tailed Student’s t-test. ns, non-significant.

**
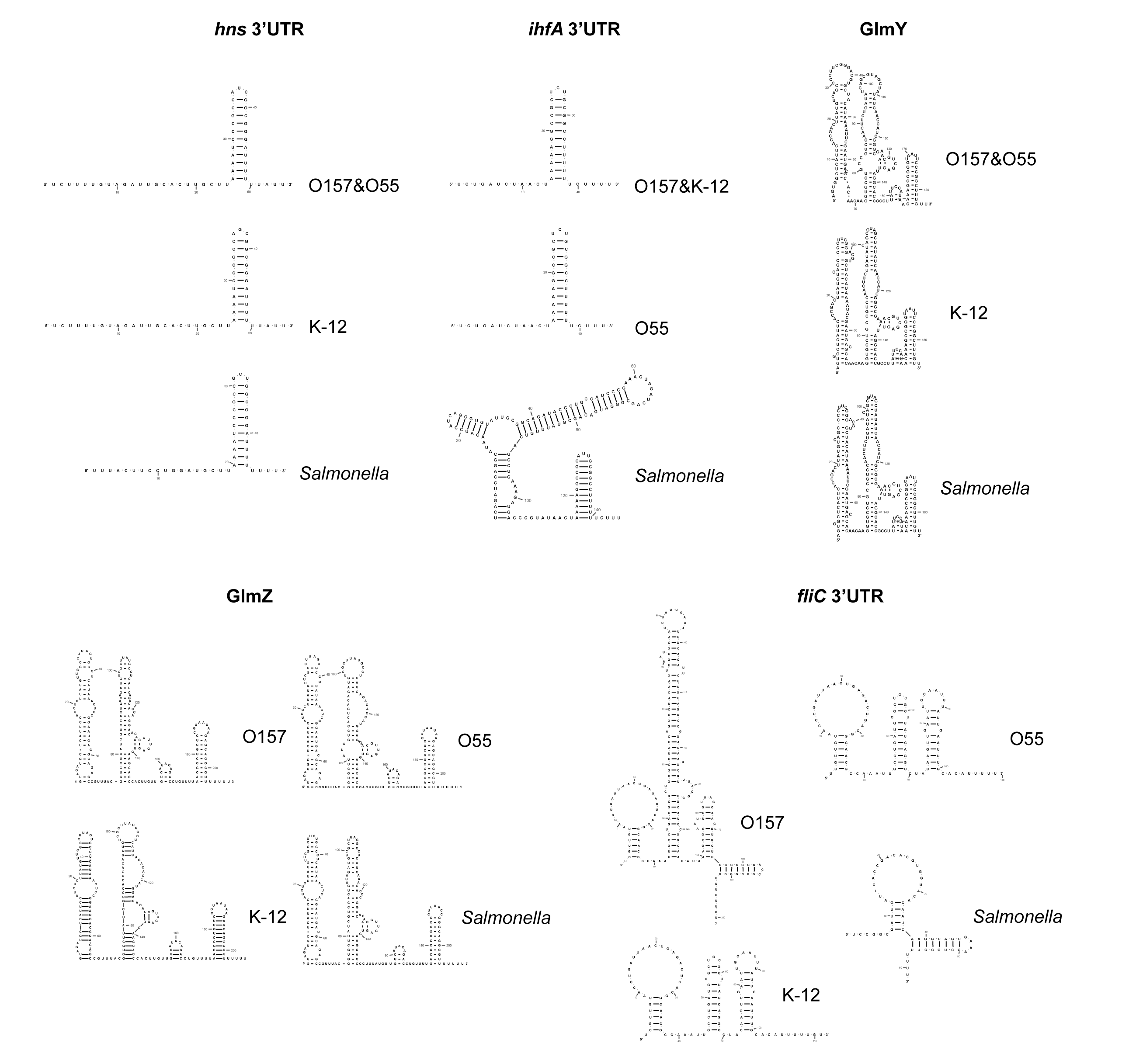
**

**Figure S5. Secondary structural comparison of *hns*, *ihfA* and *fliC* 3’UTRs and GlmY/GlmZ in the EHEC O157:H7 EDL933 strain (O157), EPEC O55:H7 CB9615 strain (O55), *E. coli* K-12 strain (K-12) and *Salmonella typhimurium* LT2 strain (*Salmonella*).**

**Supplemental Tables**

**Table S1**. **Bacterial strains used in this study.**

| **Name** | **Description** | **Source** |
| --- | --- | --- |
| WT | EHEC O157:H7 EDL933 wild-type strain | ^[5]^ |
| Δ*proQ* | EHEC O157:H7 EDL933 *proQ* deletion strain | This study |
| *proQ*-c | *proQ* complementation strain; WT transformed with  pACYC184-*proQ*; CmR, TcR | This study |
| *proQ-c** | *proQ* complementation strain; WT transformed with  pWSK29-*proQ*; ApR | This study |
| *proQ++* | *proQ* overexpression strain; WT transformed with pTrc99a-ProQ; ApR | This study |
| Δ*ler* | *ler* deletion strain | This study |
| Δ*fliC* | *fliC* deletion strain | This study |
| Δ*proQ*Δ*ler* | *ler* and *proQ* double deletion strain | This study |
| Δ*proQ*Δ*fliC* | *fliC* and *proQ* double deletion strain | This study |
| WT-*proQ*-3xFLAG | WT expressing 3×FLAG tagged ProQ at the C-terminus | This study |
| WT-P*_ler_*-*lux* | WT transformed with pMS402-P*_ler_*-*lux*; KmR | This study |
| WT-P*_hns_*-*lux* | WT transformed with pMS402-P*_hns_*-*lux*; KmR | This study |
| WT-P*_ihfA_*-*lux* | WT transformed with pMS402-P*_ihfA_-lux*; KmR | This study |
| WT-P*_fliC_*-*lux* | WT transformed with pMS402-P*_fliC_-lux*; KmR | This study |
| *∆proQ*-P*_ler_*-*lux* | ∆*proQ* transformed with pMS402-P*_ler_*-*lux*; KmR | This study |
| *∆proQ*-P*_hns_*-*lux* | ∆*proQ* transformed with pMS402-P*_hns_*-*lux*; KmR | This study |
| *∆proQ*-P*_ihfA_*-*lux* | ∆*proQ* transformed with pMS402-P*_ihfA_-lux*; KmR | This study |
| *∆proQ*-P*_fliC_*-*lux* | ∆*proQ* transformed with pMS402-P*_fliC_-lux*; KmR | This study |
| BL21 (DE3) | Strain suitable for protein purification | Novagen |
| BL21-pET28a-ProQ | BL21 transformed with pET29a-*proQ*; KmR | This study |
| BL21-pET28a-EspB | BL21 transformed with pET29a-*espB*; KmR | This study |
| EHEC O26:H11 | EHEC O26:H11 CDC 03-3014 wild-type strain | Microbiologics |
| EHEC O26:H11 ∆*proQ* | EHEC O26:H11 CDC 03-3014 *proQ* deletion strain | This study |
| EPEC O55:H7 | EPEC O55:H7 CB9615 wild-type strain | ^[6]^ |
| EPEC O55:H7 ∆*proQ* | EPEC O55:H7 CB9615 wild-type strain *proQ* deletion strain | This study |

**Table S2**. **Plasmids used in this study.**

| **Name** | **Description** | **Source** |
| --- | --- | --- |
| pKD46 | Red recombination plasmid, AmpR | ^[7]^ |
| pKD4 | Containing a kanamycin resistance cassette and the flipase recognition sites, KmR | ^[7]^ |
| pCP20 | FLP expression plasmid, AmpR, CmR | ^[7]^ |
| pACYC184 | Low copy expression vector, CmR, TcR | New England Biolabs |
| pWSK29 | Low copy expression vector, AmpR | ^[8]^ |
| pTRC99a | Expression vector, AmpR | Novagen |
| pET28a | T7 expression vector, KmR | Novagen |
| pMS402 | *lux*-based promoter reporter plasmid, KmR | ^[9]^ |
| pACYC184-*proQ* | pACYC184 carrying *proQ*, CmR | This study |
| pWSK29-*proQ* | pWSK29 carrying *proQ*, AmpR | This study |
| pMS402-P*_ler_*-*lux* | pMS402 carrying *ler* promoter fused *lux* reporter gene, KmR | This study |
| pMS402-P*_hns_*-*lux* | pMS402 carrying *hns* promoter fused *lux* reporter gene, KmR | This study |
| pMS402-P*_ihfA_*-*lux* | pMS402 carrying *ihfA* promoter fused *lux* reporter gene, KmR | This study |
| pMS402-P*_fliC_*-*lux* | pMS402 carrying *fliC* promoter fused *lux* reporter gene, KmR | This study |
| pET28a-*proQ* | pET28a carrying *proQ*, KmR | This study |

**Table S3. Primers used in this study.**

| **Primer Name** | **Sequence** | **Use** |
| --- | --- | --- |
| *rrsH*_RT_F | GAAAGCGTGGGGAGCAAAC | qRT-PCR forward primer for 16S rRNA |
| *rrsH*_RT_R | ACATGCTCCACCGCTTGTG | qRT-PCR reverse primer for 16S rRNA |
| *ler*_RT_F | CAGGAAGCAAAGCGACTG | qRT-PCR forward primer for *ler* |
| *ler*_RT_R | ACCAGGTCTGCCCTTCTT | qRT-PCR reverse primer for *ler* |
| *escT*_RT_F | GCAATAGATGCGGCTGGAC | qRT-PCR forward primer for *escT* |
| *escT*_RT_R | TCGGCTTGTAATGGTAATATCTCG | qRT-PCR reverse primer for *escT* |
| *escC*_RT_F | GACCAAAATGTTGTCGTCCCA | qRT-PCR forward primer for *escC* |
| *escC*_RT_R | AGGTTACCGCTTCGCTCG | qRT-PCR reverse primer for *escC* |
| *escN*_RT_F | AGGTTTTCTTGTTGCCTTTTGA | qRT-PCR forward primer for *escN* |
| *escN*_RT_R | TCTCCATTGGTCTGCCTATGC | qRT-PCR reverse primer for *escN* |
| *espB*_RT_F | AAAACTCCTCGGCAAGATGG | qRT-PCR forward primer for *espB* |
| *espB*_RT_R | AATAATCCCGCCAACCAAAG | qRT-PCR reverse primer for *espB* |
| *eae*_RT_F | GACGGTAGTTCACTGGACTTCTT | qRT-PCR forward primer for *eae* |
| *eae*_RT_R | TCGCCACCAATACCTAAACG | qRT-PCR reverse primer for *eae* |
| *tir*_RT_F | AAAGCAGCAGGCGAAGAGG | qRT-PCR forward primer for *tir* |
| *tir*_RT_R | TCGGCACCTGCGAATCAT | qRT-PCR reverse primer for *tir* |
| *hns*_RT_F | CAGCTGGAGTACGGCCCTGGC | qRT-PCR forward primer for *hns* |
| *hns*_RT_R | ATCCGTACTCTTCGTGCGCAGG | qRT-PCR reverse primer for *hns* |
| *ihfA*_RT_F | TCGTCTTTGGGCGAAGCGTT | qRT-PCR forward primer for *ihfA* |
| *ihfA*_RT_R | CGCTTACAAAAGCTGAAATG | qRT-PCR reverse primer for *ihfA* |
| *ihfB*_RT_F | TCGCACATTCCCGCCAAGAC | qRT-PCR forward primer for *ihfB* |
| *ihfB*_RT_R | CGCGATCGCGCAGTTCTTTA | qRT-PCR reverse primer for *ihfB* |
| *adhE*_RT_F | ATGCATCTTCAGACAGTTTATC | qRT-PCR forward primer for *adhE* |
| *adhE*_RT_R | CGCACAAACTGGGTTCCCAGT | qRT-PCR reverse primer for *adhE* |
| EvrS_RT_F | GGAGGGGCAATCAGCGAG | qRT-PCR forward primer for EvrS |
| EvrS_RT_R | GGAGAGGGGCTGGAGAGT | qRT-PCR reverse primer for EvrS |
| MavR_RT_F | TCAACGGGTGAATATGGGAAA | qRT-PCR forward primer for MavR |
| MavR_RT_R | CTATACCTGTTGAATTTGA | qRT-PCR reverse primer for MavR |
| DicF_RT_F | TGTCAGGCAACCGTATTCACCGTGAGTGGTTTTCTGGT | qRT-PCR forward primer for DicF |
| DicF_RT_R | CGTCAGATGTCCGAGTAGAGGGGGAACGGCGGACGAGACGGCGGG | qRT-PCR reverse primer for DicF |
| GlmY_RT_F | TGTCAGGCAACCGTATTCACCGTGAGTGGTAGTGGCTCA | qRT-PCR forward primer for GlmY |
| GlmY_RT_R | CGTCAGATGTCCGAGTAGAGGGGGAACGGCGTAAGGGCCGTTTCTT | qRT-PCR reverse primer for GlmY |
| GlmZ_RT_F | TGTCAGGCAACCGTATTCACCGTGAGTGGTGTAGATGCT | qRT-PCR forward primer for GlmZ |
| GlmZ_RT_R | CGTCAGATGTCCGAGTAGAGGGGGAACGGCGTTCTCGTCCGCAAAAAAA | qRT-PCR reverse primer for GlmZ |
| MP-F | TGTCAGGCAACCGTATTCACC | universal nested qRT-PCR forward primer for DicF, GlmY and GlmZ |
| MP-R | CGTCAGATGTCCGAGTAGAGG | universal nested qRT-PCR reverse primer for DicF, GlmY and GlmZ |
| *nleA*_RT_F | AGCCACTACTTCGACGGTAACC | qRT-PCR forward primer for *nleA* |
| *nleA*_RT_R | ACGAACCACTTGAGCTGTTAATCC | qRT-PCR reverse primer for *nleA* |
| *gadA*_RT_F | TCGTCGCGGCTTCGAA | qRT-PCR forward primer for *gadA* |
| *gadA*_RT_R | TGAGATATTTCAGGGAGGCTTTG | qRT-PCR reverse primer for *gadA* |
| *tnaA*_RT_F | GTACCGTGCGTAACGTCTATATC | qRT-PCR forward primer for *tnaA* |
| *tnaA*_RT_R | TCGGACCAACTTCTTCAATACC | qRT-PCR reverse primer for *tnaA* |
| *csgD*_RT_F | GCCTGAAGATTACCCGTACC | qRT-PCR forward primer for *csgD* |
| *csgD*_RT_R | TTGATCCTCCATGGCATAAA | qRT-PCR reverse primer for *csgD* |
| *flhD*_RT_F | ATACCTCCGAGTTGCTGAAACA | qRT-PCR forward primer for *flhD* |
| *flhD*_RT_R | TTTGCTGGAGATCGTCAACG | qRT-PCR reverse primer for *flhD* |
| *fliA*_RT_F | GTTAAAGTGCGGCATTTACTG | qRT-PCR forward primer for *fliA* |
| *fliA*_RT_R | GCACGGCGATAGCATCGAA | qRT-PCR reverse primer for *fliA* |
| *flgM*_RT_F | AGTCTTGCTGCGCTTCGTTGA | qRT-PCR forward primer for *flgM* |
| *flgM*_RT_R | GAGTATTGATCGCACTTCG | qRT-PCR reverse primer for *flgM* |
| *fliC*_RT_F | AGAGACAGAACCTGCTGCGGT | qRT-PCR forward primer for *fliC* |
| *fliC*_RT_R | GCTGTAAATGTGAACTCCGCG | qRT-PCR reverse primer for *fliC* |
| *prc*_RT_F | GCTATTGCAAGCAGGCCAGCT | qRT-PCR forward primer for *prc* |
| *prc*_RT_R | GCTGCGGTCAAGGTTATAA | qRT-PCR reverse primer for *prc* |
| *proQ*_del_F | ACCGTAAAGGTAACGCCAGCTCGAAGTGTAGAGACGTAATAGGCTGGAGCTGCTTC | forward primer for *proQ* deletion |
| *proQ*_del_R | TGCGCTTGTTGGCTACGTCCGTTGTAATCAGGAAATTTCATGGGAATTAGCCATGGTCC | reverse primer for *proQ* deletion |
| *proQ*_veri_F | ATCAACGGATAACGTAGCAT | forward primer for *proQ* deletion verification |
| *proQ*_veri_R | TTGCGTTCGCGACGCGGTG | reverse primer for *proQ* deletion verification |
| *proQ*_oe_F | CCCGAATTATGGAAAATCAACCTAAGTT | forward cloning primer for *proQ* overexpression |
| *proQ*_oe_R | CGCGGATCTCAGAACACCAGGTGTTCTGC | reverse cloning primer for *proQ* overexpression |
| *proQ*_com_F | CGAAGCTTATGGAAAATCAACCTAAGTT | forward cloning primer for *proQ* complementation on pACYC184 vector |
| *proQ*_com_R | CCGATATCTCAGAACACCAGGTGTTCTGC | reverse cloning primer for *proQ* complementation on pACYC184 vector |
| *proQ*_com^*^_F | CCCCCGCGGATGGAAAATCAACCTAAGTT | forward cloning primer for *proQ* complementation on pWSK29 vector |
| *proQ*_com^*^_R | CCCGGATCCTCAGAACACCAGGTGTTCTGC | reverse cloning primer for *proQ* complementation on pWSK29 vector |
| *proQ*_pET-F | CATGCCATGGCAATGGAAAATCAACCTAAGTT | forward cloning primer for *proQ* purification |
| *proQ*_pET-R | CGGGATCCTCAGAACACCAGGTGTTCTGC | reverse cloning primer for *proQ* purification |
| *espB*_pET-F | CATGCCGTATTATCAATAGTATTCAT | forward cloning primer for *espB* purification |
| *espB*_pET-R | CGGGATTCGGGTCGCTTAGCTGGGTTA | reverse cloning primer for *espB* purification |
| *ler*-lux_F | CCCGTCGAGGCTTGGTTTTTATTCTGTTTTATTTGT | forward primer to insert *ler* promoter in front of *lux* reporter gene |
| *ler*-*lux*_R | CCCGGATCCACATCTATTTCATCAAACAACCACC | reverse primer to insert *ler* promoter in front of *lux* reporter gene |
| *hns*-*lux*_F | CCCGTCGAGTATGAAAAGATATTTATTG | forward primer to insert *hns* promoter in front of *lux* reporter gene |
| *hns*-*lux*_R | CCCGGATCCAGGTAATAATAGAGCCTTAA | reverse primer to insert *hns* promoter in front of *lux* reporter gene |
| *ihfA*-*lux*_F | CCCGTCGAGTTGTAAGCGCCATAGGTTCA | forward primer to insert *ihfA* promoter in front of *lux* reporter gene |
| *ihfA*-*lux*_R | CCCGGATCCCTCGAAGAAGARGAGATTG | reverse primer to insert *ihfA* promoter in front of *lux* reporter gene |
| *fliC*-*lux*_F | CCCGTCGAGGGTTGTTTTACGACAGACGA | forward primer to insert *fliC* promoter in front of *lux* reporter gene |
| *fliC*-*lux*_R | CCCGGATCCGATTCGTTATCCTATATT | reverse primer to insert *fliC* promoter in front of *lux* reporter gene |
| *ler*_del_F | TTCCAGCTCAGTTATCGTTATCATTTAATTATTTCATGGTGTAGGCTGGAGCTGCTTC | forward primer for *ler* deletion |
| *ler*_del_R | CTTCCTGATAAGGTCGCTAATAGCTTAAAATATTAAAGCATGGGAATTAGCCATGGTCC | reverse primer for *ler* deletion |
| *ler*_veri_F | TTATTTCTTGTTTGGCTCAC | forward primer for *ler* deletion verification |
| *ler*_veri_R | ATTGTTGGTCCTTCCTGAT | reverse primer for *ler* deletion verification |
| *fliC*_del_F | TGCAGCAGAGACAGAACCTGCTGCGGTACCTGGTTAGCTTAGGCTGGAGCTGCTTC | forward primer for *fliC* deletion |
| *fliC*_del_R | AGCCTCTCGCTGATCACTCAAAATAATATCAACAAGAACATGGGAATTAGCCATGGTCC | reverse primer for *fliC* deletion |
| *fliC*_veri_F | TCAACTTGTAGGCCTGATAA | forward primer for *fliC* deletion verification |
| *fliC*_veri_R | CGACGGGTGGAAACCCAATA | reverse primer for *fliC* deletion verification |
| *ler*_IVT | TAATACGACTCACTATAGATGCGGAGATTATTTATTATGAATATGGAAAATAATTCACATACAACAAGTCCATACATTCAGCTTATAGAGCAAATTGCAGTTCTACAGCAGGAAGCAAAGCGACTGCGAGAGCAGGAAGTTCAAAGTGTAATTGAGTCGATTCAGAAGCAGATTACTTATTACAATATAACCTTACAAGAGCTGGGATATACTAATGTGCCTGATGATGGACTCGCTCGCCGGAACTCATCGAAAGGTGTTTACTACCGCAATGAAGAAGGGCAGACCTGGTCGGGCGTAGGCCGACAGCCACGCTGGCTTAAAGAAGCACTGTTGAATGGAATGAAGAAAGAAGATTTTCTTGTGAAGGACACTGAAGAAGAAATAATACCGCTGAAAAATATTTAACATGAAATAATTAA | synthesized DNA template for *in vitro* transcription of *ler* |
| *hns*_IVT | TAATACGACTCACTATAGACCTGGACTGGCCAGGGCCGTACTCCAGCTGTAATCAAAAAAGCAATGGATGAGCAAGGTAAATCCCTCGACGATTTCCTGATCAAGCAATAATCTTTTGTAGATTGCACTTGCTTAAAATCCCGCCATCGGCGGGATTTTTTATT | synthesized DNA template for *in vitro* transcription of *hns* |
| *hns*_*mut*_IVT | TAATACGACTCACTATAGACCTGGACTGGCCAGGGCCGTACTCCAGCTGTAATCAAAAAAGCAATGGATGAGCAAGGTAAATCCCTCGACGATTTCCTGATCAAGCAATAATCTTTTGTAGATTGCACTTGCTTAAAATCCCGCCATCGGCCCCTTTTTTTATT | synthesized DNA template for *in vitro* transcription of *hns_mut* |
| *ihfA*_IVT | TAATACGACTCACTATAGCCGGGCAGAAGTTAAAAAGCCGGGTCGAAAACGCTTCGCCCAAAGACGARTAATCTGATCTAACTAAAAARGCCGCTCTGCGGCCTTTTTTCTTTTACACTGTCGAAGAGTCACCGTAAAATCAACGCCATGACACTTCAGCAGAACGGATACC | synthesized DNA template for *in vitro* transcription of *ihfA* |
| *ihfA*_*mut*_IVT | TAATACGACTCACTATAGCCGGGCAGAAGTTAAAAAGCCGGGTCGAAAACGCTTCGCCCAAAGACGARTAATCTGATCTAACTAAAAARGCCGCTCTGCCCGGTTTTTTCTTTTACACTGTCGAAGAGTCACCGTAAAATCAACGCCATGACACTTCAGCAGAACGGATACC | synthesized DNA template for *in vitro* transcription of *ihfA_mut* |
| *fliC*_IVT | TAATACGACTCACTATAGCGTTGTAACCTGATTAACTGAGACTGACGGCAACGCCAAATTGCCTGATGCGCTGCGCTTATCAGGCCTACAAGTTGAATTGCAATTTATTGAATTTGCACATTCTTGTAGGCCGGATAAGGCGTTTACGCCGCATCCGGCAACATAAAGCGCAATTTGTTAGCAACGTGCTTCCCCGCCACCGGCGGGGTTTTTTCT | synthesized DNA template for *in vitro* transcription of *fliC* |
| *fliC*_*mut1*_IVT | TAATACGACTCACTATAGCGTTGTAACCTGATTAACTGAGACTGACGGCAACGCCAAATTGCCTGATGCGCTGCGCTTATCAGGCCTACAAGTTGAATTGCAATTTATTGAATTTGCACATTCTTGTAGGCCGGATAAGGCGTTTACGCCGCATCCGGCAACATAAAGCGCAATTTGTTAGCAACGACGATCCCCGCCACCGGCGGGGTTTTTTCT | synthesized DNA template for *in vitro* transcription of *fliC_mut1* |
| *fliC*_*mut2*_IVT | TAATACGACTCACTATAGCGTTGTAACCTGATTAACTGAGACTGACGGCAACGCCAAATTGCCTGATGCGCTGCGCTTATCAGGCCTACAAGTTGAATTGCAATTTATTGAATTTGCACATTCTTGTAGGCCGGATAAGGCGTTTACGCCGCATCCGGCAACATAAAGCGCAATTTGTTAGCAACGTGCTTCCCCGCCACCGCGCCGGTTTTTTCT | synthesized DNA template for *in vitro* transcription of *fliC_mut2* |
| GlmY_IVT | TAATACGACTCACTATAGAGTGGCTCATTCACCGACTTATGTCAGCCCCTTCGGGACGTGCTACATAAAATTCGAATGACGCACAACAAGGTGCCTGCCGTCCAACTTCTGATATCAGCGTAGCTATATCAACCATCGGGCGAAACGTCGAGTTAGGCACCGCCTTATTCCATAACAAAGCCGGGTAATTCCCGGCTTTGTT | synthesized DNA template for *in vitro* transcription of GlmY |
| *GlmY*_*mut1*_IVT | TAATACGACTCACTATAGAGTGGCAGTAACACCGACTTATGTCAGCCCCTTCGGGACGTGCTACATAAAATTCGAATGACGCACAACAAGGTGCCTGCCGTCCAACTTCTGATATCAGCGTAGCTATATCAACCATCGGGCGAAACGTCGAGTTAGGCACCGCCTTATTCCATAACAAAGCCGGGTAATTCCCGGCTTTGTT | synthesized DNA template for *in vitro* transcription of *GlmY_mut1* |
| *GlmY*_*mut2*_IVT | TAATACGACTCACTATAGAGTGGCTCATTCACCGACTTATGTCAGCCCCTTCGGGACGTGCTACATAAAATTCGAATGACGCACAACAAGGTGCCTGCCGTCCAACTTCTGATATCAGCGTAGCTATATCAACCATCGGGCGAAACGTCGAGTTACCGTGCGCCTTATTCCATAACAAAGCCGGGTAATTCCCGGCTTTGTT | synthesized DNA template for *in vitro* transcription of *GlmY_mut2* |
| *GlmY*_*mut3*_IVT | TAATACGACTCACTATAGAGTGGCTCATTCACCGACTTATGTCAGCCCCTTCGGGACGTGCTACATAAAATTCGAATGACGCACAACAAGGTGCCTGCCGTCCAACTTCTGATATCAGCGTAGCTATATCAACCATCGGGCGAAACGTCGAGTTAGGCACCGCCTTATTCCATAACAAAGCCGGGTAATTCGGCCGTTTGTT | synthesized DNA template for *in vitro* transcription of *GlmY_mut3* |
| GlmZ_IVT | TAATACGACTCACTATAGGTAGATGCTCATTCCATCTCTTATGTTCGCCTTAGTGCCTCATAAACTCCGGAATGACGCAGAGCCGTTTACGGTGCTTATCGTCCACTGACAGATGTCGCTTATGCCTCATCAGACACCATGGACACAACGTTGAGTGAAGCACCCACTTGTTGTCATACAGACCTGTTTTAACGCCTGCTCCGTAATAAGAGCAGGCGTTTTTTTA | synthesized DNA template for *in vitro* transcription of GlmZ |
| *GlmZ*_*mut1*_IVT | TAATACGACTCACTATAGGTAGATGCTGTAAGCATCTCTTATGTTCGCCTTAGTGCCTCATAAACTCCGGAATGACGCAGAGCCGTTTACGGTGCTTATCGTCCACTGACAGATGTCGCTTATGCCTCATCAGACACCATGGACACAACGTTGAGTGAAGCACCCACTTGTTGTCATACAGACCTGTTTTAACGCCTGCTCCGTAATAAGAGCAGGCGTTTTTTTA | synthesized DNA template for *in vitro* transcription of *GlmZ_mut1* |
| *GlmZ*_*mut2*_IVT | TAATACGACTCACTATAGGTAGATGCTCATTCCATCTCTTATGTTCGCCTTAGTGCCTCATAAACTCCGGAATGACGCAGAGCCGTTTACGGTGCTTATCGTCCACTGACAGATGTCGCTTATGCCTCATCAGACACCATGGACACAACGTTGAGTGATCGTGCCACTTGTTGTCATACAGACCTGTTTTAACGCCTGCTCCGTAATAAGAGCAGGCGTTTTTTTA | synthesized DNA template for *in vitro* transcription of *GlmZ_mut2* |
| *GlmZ*_*mut3*_IVT | TAATACGACTCACTATAGGTAGATGCTCATTCCATCTCTTATGTTCGCCTTAGTGCCTCATAAACTCCGGAATGACGCAGAGCCGTTTACGGTGCTTATCGTCCACTGACAGATGTCGCTTATGCCTCATCAGACACCATGGACACAACGTTGAGTGAAGCACCCACTTGTTGTCATACAGACCTGTTTTAACGCCTGCTCCGTAATAAGACGTCCCGTTTTTTTA | synthesized DNA template for *in vitro* transcription of *GlmZ_mut3* |

**References**

[1] Garcia-Martin, J. A.; Clote, P.; Dotu, I. RNAiFold: A Web Server for RNA Inverse Folding and Molecular Design. *Nucleic Acids Res*, **2013**, *41* (W1), W465–W470. https://doi.org/10.1093/NAR/GKT280.

[2] Gruber, A. R.; Bernhart, S. H.; Lorenz, R. The ViennaRNA Web Services. *Methods in Molecular Biology*, **2015**, *1269*, 307–326. https://doi.org/10.1007/978-1-4939-2291-8_19.

[3] McCann, H.; Meade, C. D.; Williams, L. D.; Petrov, A. S.; Johnson, P. Z.; Simon, A. E.; Hoksza, D.; Nawrocki, E. P.; Chan, P. P.; Lowe, T. M.; et al. R2DT: A Comprehensive Platform for Visualizing RNA Secondary Structure. *Nucleic Acids Res*, **2025**, *53* (4). https://doi.org/10.1093/NAR/GKAF032.

[4] Johnson, P. Z.; Simon, A. E. RNAcanvas: Interactive Drawing and Exploration of Nucleic Acid Structures. *Nucleic Acids Res*, **2023**, *51* (W1), W501–W508. https://doi.org/10.1093/NAR/GKAD302.

[5] Wells, J. G.; Davis, B. R.; Wachsmuth, I. K.; Riley, L. W.; Remis, R. S.; Sokolow, R.; Morris, G. K. Laboratory Investigation of Hemorrhagic Colitis Outbreaks Associated with a Rare Escherichia Coli Serotype. *J Clin Microbiol*, **1983**, *18* (3), 512–520. https://doi.org/10.1128/JCM.18.3.512-520.1983.

[6] Zhou, Z.; Li, X.; Liu, B.; Beutin, L.; Xu, J.; Ren, Y.; Feng, L.; Lan, R.; Reeves, P. R.; Wang, L. Derivation of Escherichia Coli O157:H7 from Its O55:H7 Precursor. *PLoS One*, **2010**, *5* (1), e8700. https://doi.org/10.1371/JOURNAL.PONE.0008700.

[7] Datsenko, K. A.; Wanner, B. L. One-Step Inactivation of Chromosomal Genes in Escherichia Coli K-12 Using PCR Products. *Proc Natl Acad Sci U S A*, **2000**, *97* (12), 6640–6645. https://doi.org/10.1073/pnas.120163297.

[8] Murray, G. L.; Attridge, S. R.; Morona, R. Altering the Length of the Lipopolysaccharide O Antigen Has an Impact on the Interaction of Salmonella Enterica Serovar Typhimurium with Macrophages and Complement. *J Bacteriol*, **2006**, *188* (7), 2735–2739. https://doi.org/10.1128/JB.188.7.2735-2739.2006/ASSET/0A693F91-FFC8-4EB8-B979-71626984FF79/ASSETS/GRAPHIC/ZJB0070655990002.JPEG.

[9] Fan, L.; Wang, T.; Hua, C.; Sun, W.; Li, X.; Grunwald, L.; Liu, J.; Wu, N.; Shao, X.; Yin, Y.; et al. A Compendium of DNA-Binding Specificities of Transcription Factors in Pseudomonas Syringae. *Nature Communications 2020 11:1*, **2020**, *11* (1), 1–11. https://doi.org/10.1038/s41467-020-18744-7.
